# Memory distrust and imagination inflation: A registered report

**DOI:** 10.1371/journal.pone.0327638

**Published:** 2025-08-01

**Authors:** Iwona Dudek, Romuald Polczyk

**Affiliations:** 1 Doctoral School in the Social Sciences, Jagiellonian University, Kraków, Poland; 2 Institute of Psychology, Faculty of Philosophy, Jagiellonian University, Kraków, Poland; Bournemouth University, UNITED KINGDOM OF GREAT BRITAIN AND NORTHERN IRELAND

## Abstract

Imagination inflation occurs when the subjective confidence of a person that an event has occurred increases after they imagine it occurring. In this project, our primary aim was to test whether memory distrust is related to the imagination inflation effect in people who are aware of the discrepancies between their own memories and what they have imagined. Our secondary purpose was to investigate whether the influence of memory distrust on imagination inflation is moderated by traits that are described as disengagement from reality and to test whether memory distrust mediates the relationship between self-esteem and imagination inflation. In a three-step procedure, participants (*N* = 279) assessed their confidence that a list of childhood events occurred to them; then they imagined three of these events and reassessed their confidence. Half of the participants were subjected to a memory distrust induction procedure. To sensitize participants to discrepancies between actual childhood memories and imagined ones, some of them received cues about the source and/or perspective of the imagined events. Memory distrust as an individual trait was found to be unrelated to the imagination inflation effect. Furthermore, the expected effect of memory distrust as a state on susceptibility to the imagination inflation effect in groups sensitized to discrepancies was not confirmed. Therefore, it seems that people who we consider to be distrustful of their memory are no more susceptible to this type of memory distortion than memory trusters.

## Introduction

Having spent months in therapy, Sheri J. Storm remembered being sexually abused by her father as a child and being forced to engage in bestiality and satanic rituals. It turned out that she was not the only person who remembered similar childhood events under the care of the same therapist. After some time, she realized that the abuse had never happened [1]. Sheri J. Storm’s therapy focused on the revelation of alleged traumatic childhood events that had been repressed from consciousness because of their harrowing nature. The theory of repressed memories has little scientific basis because no studies have provided strong support for its validity [2,3]. Unfortunately, in the course of therapy, many therapists engage clients in exercises such as repeatedly imagining scenarios (often suggested by the therapist) that might have happened [4,5].

Today, it is known that it is possible to create false memories of entire events in people just by asking them to imagine these events. Imagination enhances confidence that the imagined event occurred in real life. This effect is called imagination inflation [6] and can be classified as a subtype of confabulation that occurs when individuals assert and genuinely believe that an event occurred, despite it never actually taking place [7]. A typical procedure for studying this that was developed by Garry et al. [6] is as follows: participants are presented with the Life Events Inventory (LEI), which consists of 40 childhood events; on an 8-point scale, they are then asked to rate the likelihood of these events. Two weeks later, participants imagine four of the eight target events that are rated as low-likelihood events. They are then presented with a cover story that their LEI sheets have been misplaced, and they are asked to complete the LEI a second time. For the target events, the fact that participants had imagined them inflated their confidence that these events had occurred in their childhood. Several subsequent experiments replicated these results, e.g., [8–11].

Not only during recovered memory therapy, but also during interrogation, people can be exposed to the influence of suggestion, which can result in confabulation. This is illustrated by the case of Paul Ingram, who confessed to sexually abusing his daughters and believed that he was the leader of a satanic sect. Paul Ingram was forced to admit that his daughters would not lie about sexual abuse, and he was convinced that his memories of the horrific acts he had allegedly committed could have been repressed. His trust in his own memory was undermined. Ingram began to confess, but without giving details of the crimes. As a result of visualizing the scenes suggested by the detectives, he gave details of crimes that never really happened (detailed description of this case: [12,13]).

Memory distrust is known to increase susceptibility to suggestion. This term refers to a condition in which individuals experience a deep sense of doubt or skepticism towards their own memory. As a consequence, they become highly vulnerable to depending on external cues and suggestions as a means of shaping their beliefs or memories [14]. Two types of memory distrust are distinguished: trait and state [15]. Trait memory distrust is stable over time and refers to habitual distrust of one’s own memory ( [16,17]. It has two aspects. The first, which used to be the main focus in research on the role of memory distrust in the development of various forms of memory distortions (e.g., [15,18,19]), pertains to the subjective belief of an individual that they have a tendency to make omission errors, namely the apprehension of forgetting something [17]. The second aspect, which is moderately correlated with the first, relates to the subjective belief of an individual that they have a tendency to make commission errors, such as confusing imagination or dreams with reality [17,20].

In turn, state memory distrust is related to a specific situation in which one experiences a lack of trust in one’s own memory. In a forensic context, this situation is often related to interrogation: for example, a police officer can challenge one’s memory by subtle and manipulative questions [21,22]. Memory distrust can be also invoked because of interviewee health issues, such as alcoholism, dementia, or dissociative-related memory problems [23].

Moreover, in certain clinical cases like obsessive-compulsive disorder, individuals experience persistent doubt regarding their memories of past actions, leading them to engage in repetitive checking behaviors [24]. There are even instances of what are known as non-believed memories, where people remember an event (have a vivid recollections), but they consciously have low or no belief in it [25].

Gudjonsson claims that, under specific conditions, memory distrust can result in false confessions, especially of the pressured-internalized type, which might be accompanied by false memories of how the crime in question was committed (confabulation) [26]. He provided a detailed description of several cases in which memory distrust played a significant role in internalized false confessions, and he developed a heuristic model whereby contextual risk factors (e.g., isolation), personal vulnerability (including trait memory distrust) and acute mental states (including state memory distrust) lead to the development of memory distrust syndrome [21,22,26]. In an experimental study in which five different interrogation techniques were used, including suggesting memory problems, memory distrust was found to significantly correlate with false confession tendency [15].

Memory distrust has also been studied in the context of other subtypes of confabulation. Van Bergen et al. [27] found that individuals suffering from memory distrust are prone to accepting misinformation; however, these individuals had similar levels of interrogative suggestibility and compliance traits as those who did not suffer from memory distrust. Subjective memory ratings have been negatively correlated with compliance, but no significant correlation has been found between subjective memory ratings and false recollections (i.e., critical lures) elicited by the DRM task [28] and interrogative suggestibility [19]. Greater memory distrust is associated with a higher frequency of false memories, non-believed memories (i.e., vivid recollection of an event whose occurrence is no longer believed as much) and lower self-esteem [29].

Van Bergen and Jelicic [28] conducted an experimental study on the relationship between memory distrust and imagination inflation. First-year university students were exposed to a manipulation of memory distrust by providing feedback in a memory task that involved recalling eight events from their childhoods. Feedback was positive, negative or not provided at all and was not related to actual performance on the task. The positive feedback group was falsely informed that they had performed very well compared to other students, who usually could not even remember very much. The negative feedback group was asked why they had only been able to write down a few memories. If a participant recalled all eight events, they were asked why their memories had so few details. In addition, participants in this group were asked whether they might have had a concussion or other neurological condition that could explain their poor performance. Then, the participants were subjected to an imagination inflation procedure. This study found that manipulating memory distrust did not enhance the effect of imagination inflation.

### Overview of the present study

Although Van Bergen and Jelicic’s [30] study suggests that there is no relationship between memory distrust and susceptibility to the imagination inflation effect, in this project we still want to explore this topic. However, we assume that the relationship between memory distrust and imagination inflation will be revealed in people who are aware of the discrepancies between actual childhood memories and imagined events. In addition, our second goal is to examine the interactional effect of disengagement from reality traits [31] on the relationship between memory distrust and the imagination inflation effect. Finally, since how a person perceives memory capacity is related to how they perceive themselves, we would like to test whether self-esteem affects the magnitude of the imagination inflation effect through memory distrust. In subsequent sections, we provide detailed justification for all hypotheses.

#### Sensitization to discrepancies.

In research on another type of susceptibility to suggestion, namely the misinformation effect, participants receive misleading post-event information. According to discrepancy detection theory [32], people who are able to distinguish discrepancies between the original event and the post-event information are less susceptible (though not completely immune [33,34]) to the misinformation effect. The imagination inflation paradigm shares similarity with the misinformation paradigm to some extent: participants themselves generate misleading post-event information when they imagine childhood events. The information acquired while imagining these events increases confidence that they took place, thus causing an inflation effect. Similar to research on the misinformation effect, it has been shown that by giving people cues to help them distinguish imagined events from actual childhood events, they are less likely to experience imagination inflation [35].

By examining the misinformation effect, it is possible to check whether a participant, while answering the final memory test questions, has in memory both the original information and the information suggested in the material with which they became acquainted later [36]. It has been found that some people are aware of these discrepancies but still succumb to the misinformation [33,34]. Diagnosis of discrepancy awareness involves asking participants who have completed the memory test to state whether a particular detail, e.g., the color of a thief’s car, was mentioned in the original and/or follow-up material and to write which detail was in each piece of material (e.g., yellow car in the original material and red car in the follow-up material). A similar procedure can be used when testing another type of susceptibility to suggestion, namely interrogative suggestibility [37]. Identifying participants who are aware of the discrepancy seems to be key to studying the relationship between memory distrust and proneness to memory distortion, since only individuals who have both the original and the suggested information in memory can decide whether to rely on their own memory or the suggested information when making a decision in the post-test.

It should be noted that that there is a longstanding debate about the mechanisms of the misinformation effect; in particular, about what happens to the original memory trace. Many theories have been proposed: the parallel traces theory [38,39]; the CHARM model [40]; the fuzzy trace theory [41]; the activation-based framework [42]; retrieval-induced forgetting [43]; explanations based on the source monitoring idea (e.g., [44–51]), etc. The fact that there are “diverse routes leading to the misinformation effect” seems to be widely accepted now [52]. In the present work, we do not deny that various distortions of the memory process may moderate the impact of misinformation on memory reports. We merely want to explore some ideas which are currently less studied, namely the possibility that some participants do in fact remember (and have perfect access to) the original information as well as the misinformation contained in the post-event material, yet for some reason they give answers consistent with the latter.

In the context of imagination inflation with childhood events, it would be difficult to design a procedure to check whether participants are aware of discrepancies between actual childhood memories and imagined ones when completing the LEI post-test. Therefore, it was decided to introduce an experimental manipulation to sensitize participants to discrepancies. This manipulation was originally designed by Sharman et al. [35] to immunize against the imagination inflation effect. In Sharman et al.’s study, participants were given cues to help them resist the effect of imagination inflation. First, the subjects filled out the Life Events Inventory (LEI), thereby assessing their confidence that a list of childhood events had happened to them; then, they imagined some of these events; finally, they filled out the LEI a second time, again evaluating their confidence in the events. Participants were assigned to one of four groups. The first group received a cue about the source of their memories (they were instructed to imagine events from a first-person perspective), while the second group received a familiarity cue (a plausibility questionnaire, which contained the same events as the LEI but in a different context, before completing the LEI 2). The third group received both cues, and the fourth group received no cues. Single cues were found to be insufficient to protect against the imagination inflation effect, and only those given two cues were immune to it. This means that additional cues can sometimes protect people from becoming more confident that imagined fictitious events were genuine experiences.

This manipulation of cues used by Sharman et al. [35] is based on two postulated mechanisms of imagination inflation, one of which is source misattribution [53]. Imagination inflation appears to be caused by internal misattributions (a memory is treated as something real rather than as something imagined). As a consequence, after an imagination session, people judge the imagined event as more authentic and rate its likelihood as higher. The same mechanism is responsible for the misinformation effect, which results from erroneous external source attributions (the memory is attributed to the original event instead of to the post-event information) [31].

A cue regarding the source of a given memory seems to reduce the imagination inflation effect [35]. Such a cue may be the perspective from which childhood events are imagined. It is well established that newer memories are more often recalled from a first-person perspective than from a third-person perspective, while older memories are more often recalled from a third-person perspective than from a first-person perspective (for a review, see [54]. Thus, one can derive the assumption that the phenomenological characteristics of childhood events imagined from a third-person perspective are more likely to correspond to the phenomenological characteristics of reconstructed real childhood memories compared to those imagined from a first-person perspective [55]. As in previous research [10,35], we assume that this change in perspectives should act as a source cue that alerts participants to the differences between their imagined memories and their actual childhood memories.

The second explanation of imagination inflation assumes that familiarity can be used to evaluate the source of a memory [53] and that imagination inflation occurs as a result of misattribution of familiarity [56]. This means that more-frequently experienced events are more fluidly processed and spring more easily to mind, which can result in an increased likelihood of claiming that an event was actually experienced [57,58]. As a result of this smoother processing, there can also be an increase in confidence that an event actually occurred, i.e., there is an inflation of the imagination effect [35]. In our study, the way to sensitize participants to familiarity will be the same as in Sharman’s study: it will involve providing subjects with a source to which they can attribute their sense of familiarity of LEI events. Before completing the LEI post-test, some participants will complete a questionnaire containing the exact same events found in the LEI, but in a different context (i.e., a question about how plausible it is that a typical Pole who is about the same age as the participants had certain childhood experiences by age 10). These individuals will make a judgment based on the ease with which the events come to their mind. Giving participants a source to which they can attribute the increased cognitive accessibility of events should sensitize them to the discrepancy between their real childhood memories and imagined ones.

#### Individual differences: disengagement from reality.

Individual differences in susceptibility to imagination inflation seem to be predicted by disengagement from reality traits [31], although the research findings are inconclusive. Thus, dissociation – conceived as differences between the ability to discriminate and integrate memories, fantasies, motivations, and actions in awareness [59,60] – has been shown to correlate positively with imagination inflation [31,61,62]; however, other studies have not confirmed this [9,63]. The ability to visualize or create mental imagery positively correlates with imagination inflation [9]; however, as with dissociation, other studies have not confirmed this relationship [31,61,63]. An association between imagination inflation and hypnotic susceptibility has been found [61].

The aforementioned research was conducted in the paradigm of guided imagination inflation. However, research on false autobiographical memories and their correlates related to traits of disengagement from reality is not limited to this experimental manipulation. For example, Hyman and Billings [64] investigated the possibility of students developing false childhood memories and the role of individual differences related to the formation of these memories. Students were asked about several true childhood events (provided by a family member) and one false event (made up by the experimenter) that a family member confirmed the subject had never experienced as a child. Students described all events in two interviews. When they could not recall an event (real or false), they were encouraged to think about it and to try to imagine it. About 25% of the students created false childhood memories, and proneness to both dissociation and fantasy were found to be positively related to false memory creation. Using a similar methodology, Porter et al. [65] showed a link between the formation of false autobiographical memories and proneness to dissociation; however, in an experiment using guided visualization that was conducted by Paddock at al. [66], dissociation was not related to false memory. Horselenberg et al. [67] found a negative correlation between, on one hand, the ability to discriminate between real autobiographical events that participants themselves had documented six months earlier and, on the other hand, false events and fantasy proneness, but not dissociation, absorption, or suggestibility.

#### Memory distrust: in search of mediators of the relationship between self-esteem and susceptibility to imagination inflation.

We will test whether self-esteem affects the magnitude of the imagination inflation effect through memory distrust. Self-esteem may exacerbate memory distrust because an individual’s perceived memory capacity is related to how they perceive themselves, i.e., their self-esteem. It is difficult to clearly define the direction of this relationship. It has been shown, for example, that one predictor of older people’s perceptions of their subjective memory ratings (that is, ratings of their own memory abilities in different types of situations) is their self-esteem [68]. These authors stress, however, that perhaps this self-esteem depends on how people perceive their own memory, and the correlational nature of the research does not allow causal conclusions to be drawn. Self-esteem is negatively related to the Cognitive Failure Questionnaire (CFQ) [69], a self-report measure of memory functioning.

The sociometric theory of self-esteem assumes that self-esteem acts as a measure of an individual’s relational value in social groups [70,71], and this may explain why low self-esteem may increase memory distrust. According to this view, low self-esteem is linked to social anxiety, social alienation, and perceived maltreatment by society, among other factors [72,73]. It has been indicated that low self-esteem may result from more frequent negative feedback from others, and this may be associated with increased levels of memory distrust [29]. In addition, people with low self-esteem, who are more likely to experience rejection, may take negative feedback more easily. According to Ridley and Gudjonsson [74], they may not be able to cope with the demands of interrogation because it could make them more susceptible to developing memory distrust. Previous research has found a direct link between low self-esteem and suggestibility. Saunders [75] found that participants with low self-esteem were more likely to succumb to misinformation. On the other hand, self-affirmation techniques that rely on writing down life achievements combined with positive feedback on performance in a memory task can immunize against misinformation [76,77]. Similarly, low self-esteem has been found to increase interrogative suggestibility [78–81] (but see also: [82,83], for a review; [84]). As mentioned above, recently, in correlational studies, Zhang et al. [29] asked participants to indicate how often they experience false memory and non-believed memories. They found higher memory distrust was associated with a higher frequency of false memories, non-believed memories and lower self-esteem. In a mediation analysis, they found a significant indirect effect of self-esteem on false memory (Study 1a and Study 2) and non-believed memories (Study 2) through trait memory distrust. Also, memory distrust toward commission positively predicted the self-reported and measured occurrence of non-believed memories [85]. Interestingly, memory distrust toward commission was also positively correlated with higher recollection and belief ratings [85].

#### Hypotheses.

The aim of our study is to test hypotheses in three areas. First, we will examine the relationship between trait memory distrust (MD) and imagination inflation. Second, we will explore individual differences that might moderate the effect of MD on susceptibility to imagination inflation. Finally, we will investigate the mediating role of MD in the relationship between self-esteem and imagination inflation.

***Hypothesis 1*** concerns the relationship between the trait of memory distrust and the effect of imagination inflation. Our predictions are different for each of the two aspects of memory distrust. In terms of the subjectively experienced susceptibility to make memory omission errors, we predict that there will be no correlation between them and imagination inflation because previous research has shown that this aspect seems to correlate weakly or not at all with the formation of memory distortions (e.g., no significant correlation between trait MD and interrogative suggestibility and false recall [18,19]) (***Hypothesis 1a***). In contrast, we assume that subjectively experienced susceptibility to make commission errors will be positively correlated with imagination inflation (***Hypothesis 1b***) because errors of commission are more closely linked to the development of false beliefs and false memories than errors of omission [21]. A recent study shows that when compared to the individually perceived inclination to commit memory omission errors, individually perceived inclination to commit commission errors proved to be a better predictor of individuals’ ratings of autobiographical belief (i.e., individuals’ belief in the occurrence of specific autobiographical events). Specifically, individuals who were concerned about having distorted or false memories were more inclined to report events with lower belief ratings than those who were less concerned [86]. It is therefore likely that people who believe that they are susceptible to memory commission errors will rely more on imagined events, resulting in a greater imagination inflation effect.

We believe that the relationship between memory distrust and imagination inflation will be revealed by an experimental manipulation involving the induction of a transient state of MD and sensitization to discrepancies. In ***Hypothesis 2***, we assume that the moderating effect of MD should be revealed in groups that are ‘sensitized to discrepancies’ by at least one method (i.e., either source cue [first-person perspective in an imagination exercise], or familiarity cue [questionnaire asking, “How plausible is it that the typical Pole who is about your age had certain childhood experiences by age 10?”], or both cues). In these groups, we expect that participants with MD induction will experience a greater imagination inflation effect compared to those without MD induction (as revealed by the respective interaction being statistically significant).

Specifically, ***Hypothesis 2a***, which concerns source cue and familiarity cue, states that the effect of MD induction on imagination inflation will be present in conditions where at least one discrepancy sensitization manipulation (source cue and/or familiarity cue) is used, but it will not be present in the condition with no discrepancy sensitization manipulation.

The ***Hypothesis 2b***, which concerns the additive effect of source cue and familiarity cue, states that imagination inflation will be greater in the group that will be subjected to MD induction and will receive familiarity cue and source cue than in the group that will be subjected to MD induction and will receive one of these cues. We assume that providing two cues will increase the chances of participants noticing discrepancies and will sensitize them more than providing a single cue.

In ***Hypothesis 3*** we predict that the influence of MD on imagination inflation will be moderated by the tendency to disengage from reality, which is operationalized as high visual imagery vividness, tendency to use a visual cognitive style, strong imaginative-fantasy ability, high absorption, and tendency towards dissociation*.* We predict that this tendency to disengage from reality may not be beneficial for memory functioning because, in the imagination inflation paradigm, time is scheduled for participants to imagine the event. Participants with these traits may find it difficult to distinguish between imagined ideas and real memory traces, and the imagined information will be more vivid and accessible than real memories.

One of the traits addressed by this hypothesis is absorption, i.e., the disposition to become deeply involved in experiences, both real and imagined, while effectively excluding other sources of information [87,88]. It seems reasonable to assume that people with high levels of this disposition may have difficulty distinguishing between real and imagined events. Because absorption is sometimes considered a fundamental aspect of dissociation, a similar assumption is made for the latter as it is often seen as a core element in the typical dissociative process [89]. Dissociation in its non-pathological form is sometimes experienced in everyday life and manifests as deep focus and narrowed attention, resulting in less awareness of internal states or external activities [90].

The three traits relating to imagination (imagery vividness, visual cognitive style, and imagination immersion) do not appear to be the same [91]. They are defined differently: imagery vividness is usually understood as some sort of ability to create vivid mental images. For example, Tulving, McNulty, and Ozier [92] stated that imagery vividness is “the ease with which you can picture something in your mind” (p. 242). Similarly, Quilter, Band, and Miller [93] stated that “Vividness is the extent to which the client can mentally create images and experiences that are clear, sharp, and detailed” (p. 162). Imaginative involvement consists in experiencing some imagined events with an “almost total immersion in the activity, [and] with indifference to distracting stimuli in the environment” (p. 5) [94]. Cognitive style refers to the preferred style of information processing [95]. Because of these differences in meaning, we decided to include all of them as measures of some kind of disengagement from reality. All the hypotheses that relate to each of them separately will be tested.

**Fig 1:**
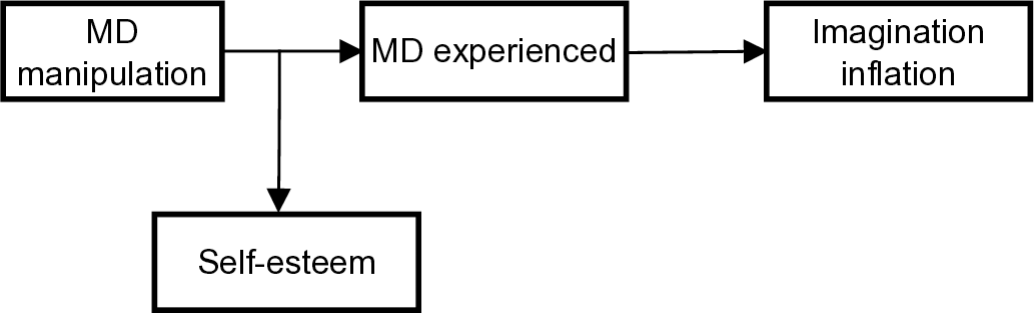
Conceptual mediation model.

Finally, in ***Hypothesis 4*** we predict that the. efficacy of memory distrust induction will be moderated by trait self-esteem in such a way that this efficacy will be higher among participants with low self-esteem. We expect this because, in the case of people with low self-esteem, any external negative feedback should have a particularly devastating effect on their self-trust, including memory distrust. After all, negative feedback just confirms what such persons believe about themselves. In contrast, people with high self-esteem may be more resistant to negative feedback (at least in the case of stable high self-esteem, perhaps less so when it is fragile). In sum, this would mean that self-esteem has a negative impact on the final variable, namely confidence that the imagined events occurred in real life, because this depends on memory distrust, which in turn depends on self-esteem as a factor moderating the efficacy of the manipulation that induces memory distrust. The proposed model is illustrated in Fig 1.

## Methods

Approval from the Research Ethics Committee of a higher education institution in Cracow, Poland was obtained (no KE/55_2022). Written consent for participation was obtained from all persons in the study prior to data collection (participants completed informed consent forms to participate in the experimental study). The recruitment period for the research participants was from 2024 February 18–2024 April 21.

### Power and sample size analysis

To identify the relevant sample sizes, a set of a priori power analyses was performed. Each of the analyses was performed for a power of 80% and alpha level of 0.05. Three analyses were performed for each problem: for the small, medium, and large effect size. Except for the analysis of the indirect effect (mediation analysis), which was based on information and tables provided by Fritz and McKinnon [96], the analyses were performed by means of G*Power 3.1 software [97]. Screenshots from G*Power are provided as supplementary materials.

The first analysis focused on the within-subject effect of imagining events on confidence rating. Repeated-measures ANOVA was chosen as the method of analysis. A rather moderate-strength correlation of 0.5 between the pre- and post-test was assumed. For the small, medium, and large effect sizes (*f* = 0.10. 0.25, and 0.40), the required sample sizes were 199, 34, and 15 respectively (it should be noted that the conventional effect sizes are for between-subjects rather than within-subjects design [98]).

The second power analysis looked at the sample sizes required to detect between- and within-subjects interactions; this is needed in the case of the hypothesis that MD induction causes a greater change in the pre- versus post-tests of imagination inflation. Again, assuming small, medium, and large effect sizes (**f* *= 0.10. 0.25, and 0.40), the sample sizes required to detect interactions, as indicated by G*Power were, 200, 34, and 16, respectively.

The third problem concerned the moderation analysis. Technically, detecting moderation in a regression analysis framework consists in analyzing whether the product of the independent variable and the moderator is a significant predictor of the dependent variable after controlling for the independent variable and the moderator. This can be analyzed as a statistically significant increase in R-square by means of G*Power. Effect sizes of *f*^2^ equaling 0.02, 0.15, and 0.35 were assumed, resulting in sample sizes of 395, 55, and 35 participants, respectively.

Finally, the power analysis for the indirect effect (mediation analysis) was based on existing tables: Fritz and MacKinnon [96] provided information about the sample sizes needed for 80% power for tests of mediation for four effect sizes concerning the predictor > mediator and mediator > dependent paths: 0.14, 0.26, 0.39 and 0.59. When both paths are close to the smallest effect size, the required sample size is 558. An effect size of 0.26 for both paths requires a sample size of 162, while an effect size of 0.39 requires a sample size of 78.

Given the resources available, we decided to use a sample size of 300 participants as this would allow us to detect at least medium-sized effects in all analyses, and large ones in some of them.

### Participants

The first part of the study involved 375 participants, of whom the final, third part was completed by 306 participants. After combining data from all three parts of the experiment and excluding data that did not meet the criteria (see Data exclusion section for details), data from 279 participants were included in the analysis. Participants were between 18 and 60 years old (M = 22.88, SD = 5.70) and there were 210 women, 65 men, three participants declared a different gender, and one declined to indicate it. These participants received 60 PLN (about 14 EUR) for their participation. Participants were recruited through posters and advertisements on social media and job sites. Previous participation in memory distortion research precluded candidates from taking part in the study.

### Design

The design was a mixed design: 2 (memory distrust induction: yes *vs.* no) × 2 (source cue: first-person perspective *vs.* third-person perspective) × 2 (familiarity cue: plausibility questionnaire first *vs.* LEI 2 first) × 2 (event type: imagined vs. not imagined) × 2 (LEI completion time: LEI 1 (pre-test) *vs.* LEI 2 (post-tes). The between-subjects variables were MD induction, the perspective cue (whether the participant imagines events from a first-person or third-person perspective), and the familiarity cue (whether the participant is the first to complete the LEI 2 or the plausibility questionnaire). Participants were evenly assigned to these three between-subjects conditions: memory distrust induction: yes (*n* = 146), no (*n* = 133), perspective cue: first-person (*n* = 142), third-person (*n* = 137), and familiarity cue: plausibility first (*n* = 150), LEI 2 first (*n* = 129). The within-subjects variables were time of completion, LEI, and event type. The counterbalance was that each participant imagined a randomly selected three of the six target events. The other three events served as controls. The order of presentation of these events was counterbalanced between subjects.

### Materials

**Life Events Inventory (LEI)** consists of 20 events, translated and adapted to Polish culture by the first author; it is similar to Marsh et al.’s [55] questionnaire. Six of the events are critical target events; three events are unrealistic and are included to ensure that participants pay attention and provide credible answers. Subjects rated their confidence that the events happened before the age of 10 on a Likert scale from 1 (definitely did not happen to me prior to age 10) to 8 (definitely did happen to me prior to age 10). The final result was computed as the mean ratings of the three target events.

**The plausibility questionnaire** consists of the same events as the LEI. Participants rated “how plausible it is that the typical Pole who is about your age had certain childhood experiences by age 10” and gave their rating on a Likert scale from 1 (*not at all plausible*) to 8 (*very plausible*). As in the study by Sharman et al. [35], in order to avoid participants linking items from the LEI and plausibility scales to the personality questionnaires, the measures were presented in a completely different layout.

**The procedure for inducing state memory distrust** consists of two stages and is administered *via* computer, similarly to Dudek & Polczyk [18]. Participants were asked to recall eight life events that happened to them when they were less than 10 years old. They briefly described (1) what happened, (2) where it happened and (3) who took part in the event. This part of the procedure was based on the research of Winkielman et al. [99] and Merckelbach et al. [100], which has shown that remembering many events from childhood reduces the level of trust in one’s own memory abilities. Effort in the task is assumed to undermine one’s trust in one’s memory as participants attribute perceived difficulties during the task to the poor state of their own memory [99]. The second part of the procedure uses the premise that a key factor in MD is that giving false feedback makes people feel insecure [101]. A similar procedure has already been used by Szpitalak [102] that involves memorizing words from a list of 60 words in two minutes, then trying to remember as many words as possible. The computer screen then displayed a false message which suggests that the participant performed poorly on this task compared to other participants.

The second method of inducing MD is rooted in the Barnum effect [103], which involves most people agreeing to descriptions of their personalities that are supposedly tailored specifically to them but are in fact vague and non-specific. The computer screen displays information about the “nature of memory processes”, e.g., “It is possible that you often experience the tip-of-the-tongue phenomenon when you know that you remember something but are unable to express it”. According to the Barnum effect, the manipulation will succeed if it causes participants to begin to doubt their memories.

In the control condition, participants completed questionnaires unrelated to the purpose of the study. We decided to use a set of questionnaires measuring various kinds of susceptibility to influence, need for closure, and social desirability, which could be useful for exploratory analyses: the Gudjonsson Compliance Scale ( [104]; Polish adaptation: [105]); The Inventory of Suggestibility ( [106]; Polish adaptation: [107]); The Measure of Susceptibility to Social Influence ( [108]; Polish adaptation: [36]); the Need for Closure Scale ( [109]; Polish adaptation: [110]); and the Marlowe–Crowne Social Desirability Scale ( [111], Polish adaptation: [112]).

**Manipulation check.** Before and after the MD induction procedure, participants completed a questionnaire containing four filler questions and one critical statement: “At the moment, I trust my ability to remember things”. Participants indicated to what extent they agree with these statements (including the statement concerning memory distrust) using an analog visual scale, whose scores were used in the analysis.

#### Squire Subjective Memory Questionnaire.

(SSMQ; [113]; Polish adaptation: [114]). This scale measures an aspect of memory distrust that is related to errors of omission, i.e., forgetting past experiences [17]. This questionnaire consists of 18 items rated on a nine-point scale, with answer options ranging from “disastrous” (−4) to “perfect” (4). The scale items include “My ability to search through my mind and recall names or memories I know are there is…” and “My ability now to remember what I read and what I watch on television is…” The internal consistency of the Polish version of SSMQ (Cronbach’s alpha) is .89, and the test-retest reliability is .87. Cronbach’s alpha in this study was .88.

#### Memory Distrust Scale.

(MDS; [17]). This scale captures memory distrust in relation to commission errors, i.e., remembering experiences that did not take place in the past. This tool contains 20 items, which are rated on a Likert scale ranging from “strongly disagree” (1) to “strongly agree” (7). The scale items include “I often look for physical evidence, such as photographs, to check whether things really happened the way I remember them” and “I believe some of my memories may have originated entirely from my imagination”. The total score ranges from 20 to 140, with higher scores suggesting greater levels of memory distrust. Internal consistency in validation studies was very high (Cronbach’s alpha ranging from .95−.96), and test-retest reliability over a period of several weeks to 19 months was good. In the present study, a Polish translation of the MDS was used. The reliability of the scale (Cronbach’s alpha) was .93.

#### Rosenberg Self-Esteem Scale.

(SES; [115]; Polish adaptation: [116]. This scale measures global self-esteem and consists of 10 items (e.g., “At times I think I am no good at all”), to which participants respond on a four-point scale ranging from 1 (“I strongly agree*”*) to 4 (“I strongly disagree”). The Cronbach’s alpha of the Polish version of the SES is between .81 and .83. Its theoretical validity has been confirmed [116]. Internal reliability as measured by Cronbach’s alpha in the present study was .84.

#### Vividness of Visual Imagery Scale.

(VVIQ; [117]; Polish translation: Siuta, unpublished translation). This 16-item questionnaire measures imagery vividness. VVIQ involves having subjects imagine several suggested scenes. For each scenario, participants are asked to rate how vivid the image is using a 5-point scale, where “1” means “No image at all, I only ‘know’ I am thinking of the object”, “2” means “Dim and vague image”, “3” means “Moderately realistic and vivid”, “4” means “Realistic and reasonably vivid” and “5” means “Perfectly realistic, as vivid as real vision”. Example scenario: “Visualize a rising sun. Consider carefully the picture that comes to your mind’s eye”. An example of an item whose vividness is rated on the scale described above is “The sun rising above the horizon into a hazy sky”. Marks [117] obtained a test-retest reliability of .74 and a split-half reliability of .85. In our study the value of internal reliability as measured by Cronbach’s alpha was .89.

#### Verbalizer-Visualizer Questionnaire.

(VVQ; [118]; Polish translation: Siuta, unpublished translation). This self-report test comprises 30 questions and is designed to measure the extent to which people prefer to process information visually or verbally. Fifteen of the questions are diagnostic and refer to participants’ verbal and visual cognitive style. Each question is rated as “true” or “false”. Two dimensions are computed separately for the visual and verbal styles. An example of an item for visual ability: “My powers of imagination are better than average”). An example of an item for verbal ability: “I can easily think of synonyms for words”. The value of Cronbach’s alpha in this study was .58 for the visual scale, and also .58 for the verbal scale.

#### Inventory of Childhood Memories and Imaginings.

(ICMI; [119]; Polish adaptation: [120]). This tool measures imagination immersion. The ICMI is a 52-item true-false questionnaire that is designed to assess the vividness of imagery, fantasy proneness, and strange experiences. The ICMI includes items such as “When I was younger, I enjoyed fairy tales” and “Now, I still live in a make-believe world some of the time”. Test-retest reliability and construct validity data are adequate, as documented in Lynn and Rhue [121]. The value of Cronbach’s alpha in this study was .83.

#### Tellegen Absorption Scale.

(TAS; [88]; Polish translation: Siuta, unpublished translation). This scale measures absorption, defined as the tendency to experience states of total attentional engagement. A version consisting of 37 self-descriptive statements with which the participant can agree or disagree will be used. An example item is “I like to watch cloud shapes change in the sky”. Internal reliability as measured by Cronbach’s alpha in the present study was .85.

#### Dissociative Experiences Scale.

(DES; [122,123]. This scale measures the frequency of various interruptions and disturbances of consciousness, memory, and identity. In this study, we will use form C of the DES [124], in which participants rate how often they have each experience compared with other people. For example, participants are told “some people have the experience of finding themselves dressed in clothes that they don’t remember putting on” and are asked to “place a cross to show how much of the time this happens to you”. One end of the scale is labeled “much less than others”; the midpoint of the scale is “about the same as others”, and the other end is “much more than others”. Internal reliability as measured by Cronbach’s alpha in the present study was .91.

The total score of each of these questionnaires was used in later analyses.

### Procedure

**Fig 2:**
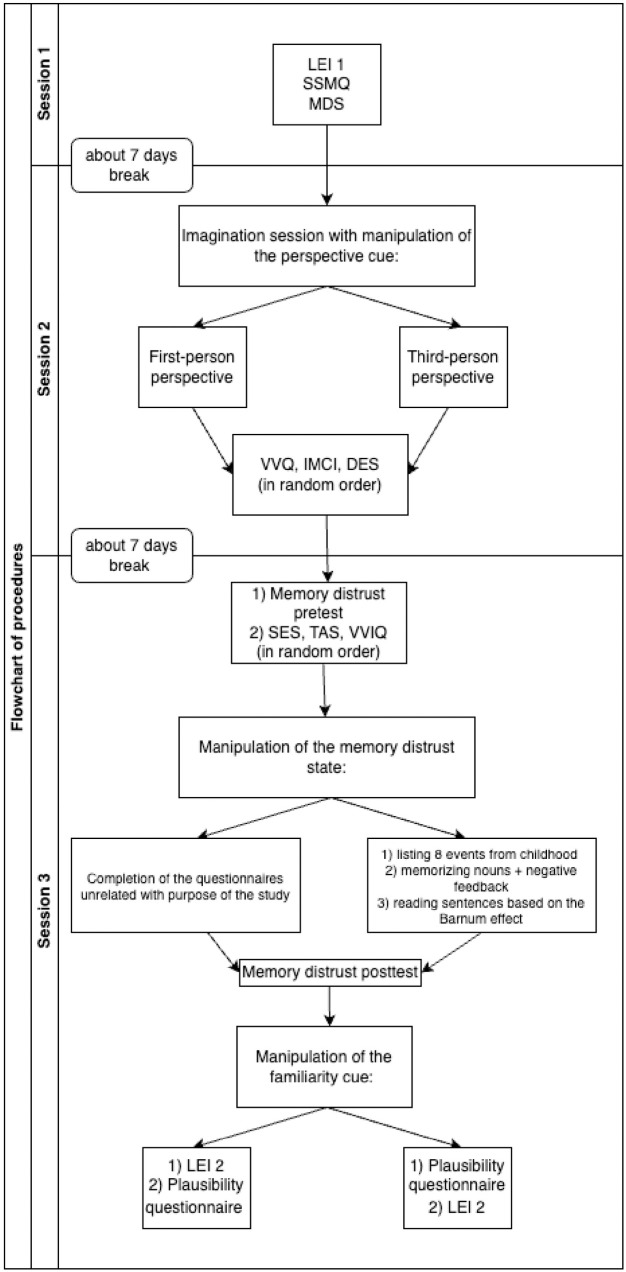
Flowchart of the experimental procedure.

The procedure consisted of three parts, as shown in Fig 2.

**Part one.** The first part took place online. Participants received a link by e-mail to an online application Qualtrics, where they completed the LEI 1 (pre-test), SSMQ and MDS questionnaires. It took approximately 10 min to complete this stage, but no time limit was imposed.

**Part two.** Approximately seven days after completing part one, participants took part in the laboratory session, individually or in groups of 2–10 people. We used a cover story similar to that of Sharman et al. (2005) [35], which states that participants will practice an imagination-based dream control technique. Each participant sat at a computer workstation separated by an opaque wall from other participants. All instructions were displayed on the computer screen. Participants imagined three of the six target items from the LEI. The other three served as controls. The target events were counterbalanced among the subjects. Based on a guided imaginary instruction used in previous studies (e.g., [125,126]), the instruction the participants had received depended on the perspective from which they imagined the events (see https://osf.io/dxf8g/ for detailed instructions). In the appropriate box on the screen, participants described what they had imagined about the event. Then filled out three personality questionnaires (VVQ, IMCI and DES) in a random order; this took about 15 minutes, after which they were released. To maintain the plausibility of the cover story, participants were given a bogus event to dream about (receiving a pet as a gift) and asked to imagine it before going to sleep at least twice before the next laboratory session.

**Part three**. Approximately seven days later, participants completed the pre-test for MD manipulation and the remaining three personality questionnaires (SES, TAS and VVIQ in random order). Then, depending on the group, participants either participated in the MD induction procedure or performed the tasks provided for the control group (in which there was no MD induction). After that, the post-test for MD manipulation was applied. Subsequently, half of the participants completed the post-test LEI (LEI 2) and then the plausibility questionnaire; the other half completed the plausibility questionnaire and then LEI 2. These tools were separated by a filler task. Finally, participants answered questions concerning the purpose of the study and whether they have previously participated in research on memory and the psychology of witness testimony. At the end, the participants were debriefed.

#### Data exclusion.

We followed the criteria outlined in the registered report protocol. Minor changes were made to some of the criteria, as noted for each.

##### LEI 1 and LEI 2 questionnaires.

(criteria based on previous research [55,127])

1. *The participant consistently rates all items as 1 or 8 on LEI 1 or LEI 2.*

One participant marked 1 in LEI 1 alone but given that it is theoretically possible that none of the LEI situations occurred to this person in childhood, and the fact that their data for all parts of the study were good quality, and the fact that they did not meet any other exclusion criteria, we decided not to exclude those data from the analyzes.

1. *The participant provides a rating other than 1 on LEI 1 or LEI 2 for any of the three unrealistic LEI events (that is, “Won a million zlotys”, “Shook hands with the President”, “Played for Wisła Kraków”).*

We decided not to consider the following event: “Shook hands with the President”, as it is quite possible that the participants have experienced this (e.g., they have shaken hands with the city president or some politician who has been president in recent years). Based on the analysis of the responses to the other two items: “Won a million zlotys.” and “Played for Wisła Kraków” we removed data from participants (*n* = 3), who marked “8” in LEI 1 for both of these items.

##### Post-experimental interview.

1. *The participant correctly guesses the purpose of the study or the research hypothesis. The researcher will make a decision based on the answer to the question ‘What is the purpose of the research in your opinion (what does it investigate)?’ It is planned to use three competent judges to determine whether what the subject said about the hypothesis is consistent with the hypothesis. Kendall’s W coefficient will be used to determine concordance among judges.*

In the analysis of this criterion, we also considered the study comment question, as some participants included their thoughts on MD manipulation in the response.

We excluded data from participants (*n* = 10):

1. Who explicitly wrote that the relationship between imagining events and later assessing how likely they were to have occurred in the participant’s life may be studied (e.g., “The effect of recalling memories on deciding about statements regarding certain events, susceptibility to this, and how imagination influences this.”, “The effect of imagination on the occurrence of false memories”);
2. Who indicated that the relationship between MD and the evaluation of the probability of occurrence of events may be under investigation (e.g., “I believe the study was designed to demonstrate the effect of suggestion on the subsequent ability to evaluate previously evaluated events, but after the suggestion has occurred. I.e., whether the impact of suggestion associated with an unfavorable outcome on my ability to remember would be noticeable on later answer marks.”);
3. Who did not succumb to the memory distrust manipulation (e.g., “I realized that the message that my score was so low was untrue, so it did not lower my trust in my memory”).

We decided to deviate from the planned Kendall’s W coefficient as an indicator of concordance among the judges because it is sensitive to ties [128], and in the present research, as much as 96% observations were ties – all judges assigned zeros, or all assigned ones. We applied the intraclass correlation coefficients (ICC) instead, calculated as the two-way random-effects model. Mean absolute agreement among judges was .88 [.86,.90], p < .01, and mean consistency was .89 [.86, .91], p < .001. In addition, we calculated Cohen’s Kappa index of agreement, for any of the three pairs of judges. The results were:.86,.69, and .68 (all *ps* < .001). Therefore, the interrater reliability can be considered satisfactory.

1. *The participant has already participated in a study on memory distortion. The researcher will make a decision based on the description of the study provided by the participant. The exact question in the interview will be “Have you ever participated in a similar study?” (Choose “yes” or “no”; if the participant selects “yes”, an additional window will appear asking for a description of what the study involved.)*

No one was excluded on the basis of this criterion.

1. *The participant reports technical problems (when asked to comment on the study procedure or during the procedure itself).*

There were no technical problems, other than an incorrectly entered ID, resulting in an inability to match parts of the survey, leading to a lost data from the participant.

1. *The participant answers “No” to the seriousness check question: “Did you give honest and truthful answers to the questions? Did you take the study survey seriously? Your answer to this question will not affect your reward for participating in the survey.”*

All participants indicated that they took participation in the experiment seriously.

##### Attention check.

*The participant fails at least one of the three attention checks. These will be items hidden among the personality questionnaire questions, such as “Please select ‘very often’ (this is to check your attention)”, with the response options from (1) never to (4) very often.*

We relaxed the criterion so that one failed attention check was acceptable, as we considered that with so many questionnaires to complete it was easy to make a mistake. Consequently, we did not remove any data (in six cases participants had two out of three passed attention checks).

##### Perspective Cue Adherence.

*The researcher will analyze the imagery descriptions generated by the participants. If the perspective does not match the one to which the person was assigned in at least one (out of three) description provided by the participant, the data from this participant will be excluded.*

We analyzed the written records of the mental images created by the participants. However, because it is difficult to determine how closely the notes coincided with what the mental images created by the participants actually looked like, we removed (*n* = 5) those who wrote that they did not complete the task at all (because, for example, these events could not have happened to them).

#### Study Termination.

We assumed that our research sample size would be 300 subjects. Due to limited financial resources for the study and difficulties in recruiting people who had not previously participated in research thematically related to the current project (e.g., on memory distrust), it was decided to terminate the study.

## Results

**Manipulation check** A two-way ANOVA with one between-subjects factor (inducing memory distrust *vs.* the control group) × one within-subjects factor (mean results on VAS regarding trusting memory before and after manipulation) was conducted. The decrease in memory trust as a result of experimental manipulation was compared between the groups with (*n* = 146) and without (*n* = 133) MD induction. In the MD induction groups, the decrease in memory trust was significant (*M* = 70.72*, SD* = 18.84 vs. *M* = 52.77, *SD* = 22.30); *F*(1,277) = 143.60, *p* < .001, η^2^ = .34, whereas no such decrease was observed in the groups where MD was not induced (*M* = 69.32, *SD* = 22.39 vs. *M* = 70.79, *SD* = 22.42; *F*(1, 277) = 0.88, *p* = .348, η^2^ = .003). The overall interaction of the pre-posttest factor and the MD groups was also significant (*F*(1, 277) = 80.16, *p* < .001, η^2 ^= .22). Then we compared the mean change in memory distrust for the MD induction group with the half of the pooled mean standard deviation, calculated using Cohen’s formula ( [96], p. 67). The mean change in memory distrust for the MD induction group was 17.95 which was greater than half the pooled standard deviation (10.31). This change in memory trust was significantly greater than one sten, which, according to our registered report protocol. As an additional analysis we calculated how many participants reported an increase of distrust [129]. There were 106 such participants in the experimental group (73%) and only 34 (25%) in the control group. Taken together, these results confirmed the effectiveness of MD manipulation.

**Inflation score** We used procedures based on those suggested by Sharman and Barnier [126]. First, we calculated the change score for each target event (imagined and not imagined) by subtracting the confidence ratings at LEI 1 from the confidence ratings at LEI 2. Then, we will subtract the change score for not imagined events from the change score for imagined events. The final score was the mean change of imagined events (*n* = 3) minus the mean change of the not imagined (**n* *= 3) events. A positive inflation score indicated imagination inflation, while a negative score indicated a decrease in confidence ratings. We termed this indicator an individual index of susceptibility to the imagination inflation effect and used it to verify Hypotheses 1 and 4.

**Hypothesis 1** To verify Hypothesis 1, we calculated correlation coefficients between two measures of MD (SSMQ, Hypothesis 1a and MDS, Hypothesis 1b) and the individual index of susceptibility to the imagination inflation effect, using the JASP software [130] with default priors. To examine Hypothesis 1a assuming the non-existence of an effect (no relationship between the SSMQ and the imagination inflation effect we specified a two-sided alternative hypothesis. Pearson’s *r* correlation was −.064 (95% CI [0.054, −0.179]) and the Bayes factor (BF_10_) for it was 0.132. According to the interpretation guidelines proposed by van Doorn et al. [131] this indicates moderate evidence in favor of the null hypothesis. Additionally, to assess the robustness of the Bayes factor to our prior specification, we have looked at how the value of the Bayes factor is changing for different prior distributions. Across a wide range of widths, except for small values of prior, the Bayes factor indicated moderate support for null hypothesis. Thus, hypothesis 1a can be considered confirmed.

To verify Hypothesis 1b, in which we assumed a positive relationship between the MDS, and the imagination inflation effect, we set one-sided alternative hypothesis. Pearson’s *r* correlation coefficient was .045 (95% CI [0.167, 0.004]) and the Bayes factor (BF_(+0)_ was .153, which indicate moderate evidence in favor of the null hypothesis. Therefore, Hypothesis 1b was not confirmed.

While testing Hypotheses 2 and 3, we decided to deviate from the mixed repeated measure ANOVA declared in Stage 1 in favor of a generalized estimation equation (GEE) approach. We decided on this change because participants had different imagined and control items. A long data format was applied and analyses were conducted using SPSS 29 [132]. Conceptually, this analysis answers the same research questions as the planned one. We tested the GEE models with an independence working correlation structure and robust standard errors in which the difference in LEI 2 and LEI 1 questionnaire scores for the critical items (i.e., the three imagined items and the three unimagined items) was used as the dependent variable. Predictors/ covariates are indicated when describing the analyses of the respective hypotheses.

**Hypothesis 2** To verify Hypothesis 2a stating that the effect of MD induction on imagination inflation effect will be present in conditions where at least one discrepancy sensitization manipulation (source cue and/or familiarity cue) is used, but it will not be present in the condition with no discrepancy sensitization manipulation, we tested the GEE model with the following predictors: a variable indicating whether MD was induced (yes/no), a variable indicating whether the item was imagined (yes/no), and a variable indicating the presence of any cues (perspective or/and familiarity cue (yes/no). There was a main effect for the predictor indicating whether the item was imagined, Wald chi^2^(1) = 23.43, *p* < .001, which confirms the occurrence of an imagination inflation effect (*M* = 0.74, *SE* = 0.10 for imagined items; *M* = 0.18, *SE* = 0.07 for not imagined items). There was no three-way interaction between the predictors that would indicate a moderating effect of MD on the relationship between receiving a cue and susceptibility to the imagination inflation effect, Wald chi^2^(1) = 0.59, *p* = .442. No other effects related to Hypothesis 2a were significant (full model is included in Supplementary materials in S1 Table. Therefore, Hypothesis 2a was not confirmed. Table 1 shows the estimated marginal means and their standard errors for each condition, along with pairwise comparisons between MD and Non-MD condition.

**Table 1:** Pairwise comparisons between conditions in the first model.

Whether the event was imagined	Presence of cue(s)	Mean difference (MD induction: MD – Non-MD)	SE	*p*
No	No	−0.24	0.26	.351
	Yes	0.24	0.15	.120
Yes	No	−0.62	0.34	.067
	Yes	0.22	0.18	.242

Note. MD = Memory distrust; Non-MD = No memory distrust; SE = Standard error.

Then we verified Hypothesis 2b concerns the additive effect of source cue and familiarity cue, which states that imagination inflation effect will be greater in the group that will be subjected to MD induction and will receive familiarity cue and source cue than in the group that will be subjected to MD induction and will receive one cue. We tested a model that differed from the one described above in that we coded the presence of a cue at three levels (none, one cue, and two cues). Here, the three-way interaction between the indication of MD induction, the number of cues, and the indication of whether the item was imagined, was nonsignificant, Wald chi^2^(2) = 0.97, *p* = .615. No other effects related to Hypothesis 2a were significant (full model is included in Supplementary materials in S2 Table. Consequently, Hypothesis 2b was also not confirmed. The difference between the MD and non-MD conditions when no cues were provided for the imagined event, as well as the difference between the MD and non-MD conditions when one cue was provided for the imagined event, were non-significant, Wald chi^2^(1) = 3.46, *p* = .063; Wald chi^2^(1) < 0.001, *p* = .988, respectively. Similarly, the difference between the MD and non-MD conditions when two cues were provided for the imagined event was non-significant, Wald chi^2^(1) = 3.59, *p* = .058. Table 2 shows the estimated marginal means and their standard errors for each condition, along with pairwise comparisons between MD and Non-MD condition.

**Table 2:** Pairwise comparisons between conditions in the second model.

Whether the event was imagined	Cue condition	Mean difference (MD induction: MD – Non-MD)	SE	*p*
No	No cue (0)	−0.24	0.26	.348
	One cue (1)	0.13	0.18	.444
	Two cues (2)	0.43	0.28	.117
Yes	No cue (0)	−0.63	0.34	.063
	One cue (1)	0.00	0.23	.988
	Two cues (2)	0.58	0.31	.058

Note. Cue condition = the number of cues provided (0 = no cue, 1 = one cue, 2 = two cues); MD = Memory distrust; Non-MD = No memory distrust; SE = Standard error

Looking at the same data the other way one can analyze whether the cues have a different impact on imagination inflation effect in the conditions with and without MD induction. Such an analysis is presented in Table 3 and 4.

**Table 3:** Effect of presence of cue(s) on imagination inflation effect in the conditions with and without MD induction.

Whether the event was imagined	MD induction group	Mean difference (Presence of cue: No – Yes)	SE	*p*
No	MD	−0.38	0.21	.069
	Non-MD	0.10	0.21	.656
Yes	MD	−0.25	0.28	.367
	Non-MD	0.58	0.27	.028

Note. MD = Memory distrust induction; Non-MD = No memory distrust induction; SE = Standard error

**Table 4:** Effect of different number of cues on imagination inflation effect in the conditions with and without MD induction.

Whether the event was imagined	MD induction group	Cue condition (A)	Cue condition (B)	Mean difference (B – A)	SE	*p*
No	MD	No cue (0)	One cue (1)	0.38	0.22	.080
			Two cues (2)	0.40	0.27	.132
		One cue (1)	Two cues (2)	0.02	0.23	.914
	Non-MD	No cue (0)	One cue (1)	−0.00	0.23	.997
			Two cues (2)	−0.28	0.27	.306
		One cue (1)	Two cues (2)	−0.28	0.24	.247
Yes	MD	No cue (0)	One cue (1)	0.11	0.29	.696
			Two cues (2)	0.46	0.34	.185
		One cue (1)	Two cues (2)	0.34	0.30	.254
	Non-MD	No cue (0)	One cue (1)	−0.52	0.28	.068
			Two cues (2)	−0.75	0.30	.012
		One cue (1)	Two cues (2)	−0.23	0.23	.319

Note. MD = Memory distrust induction; Non-MD = No memory distrust induction; Cue condition = the number of cues provided (0 = no cue, 1 = one cue, 2 = two cues); Mean difference (B – A) represents the change in confidence between these conditions within the same MD induction group and imagination condition. SE = Standard error

As can be seen in Table 3, for the imagined items in the MD induction condition, the presence of a cue/the cues did not affect the imagination inflation effect (cue(s) present: *M* = 0.77, *SE* = 0.14, cues absent: *M* = 0.52, *SE* = 0.24, *p* = .367). Similarly, the number of cues (0, 1 or 2) did not affect imagination inflation effect (see Table 4): the differences between the condition with no cue (*M* = 0.52, SE = 0.24) and one cue (*M* = 0.64, *SE* = 0.17) and two cues (*M* = 0.98, *SE *= 0.25) were non-significant (*p* = .696 and *p* = .185, respectively).

In contrast, for items imagined in the no MD induction condition, the presence of cue(s) reduced the imagination inflation effect (cue(s) present: *M* = 0.55, *SE* = 0.12, cues absent: *M* = 1.14, *SE* = 0.24, *p* = .028) (see Table 3). Looking in greater detail in Table 4 we see that the effect of the cues was found to be significant for two cues: the differences between the condition with no cue (*M* = 1.15, *SE* = 0.24) and two cues (*M* = 0.40, *SE* = 0.18) was significant (*p* = .012), but not for one cue: the differences between the condition with no cue and one cue (*M* = 0.63, *SE* = 0.15) was non-significant (*p* = .066). There was no significant difference between the condition with one and two cues (*p* = .319).

**Hypothesis 3**. In each GEE model, predictors were a variable indicating whether MD was induced (yes/no) and a variable indicating whether the item was imagined (yes/no). In each subsequent model, one of the measures of disengaging from reality was entered as a covariate. The results of the interaction effects of these measures on the relationship between MD and imagination inflation are presented in Table 5. None of the interactions was statistically significant, which means that Hypothesis 3 was not confirmed. Also, none of the measures of disengaging from reality was significant as the main effect (**p* *= .301 for TAS, **p* *= .947 for DES, *p* = .758 for ICMI, *p* = .673 for VVIQ, and for the two aspects of VVQ, *p* = .226 for the visualizer and *p* = .339 for the verbalizer).

**Table 5:** Results of moderation analysis.

Moderator	Wald Chi^2^ (3)	*p*
TAS	0.39	.942
DES	0.73	.866
ICMI	4.22	.239
VVIQ	1.03	.794
VVQ VIS	1.12	.772
VVQ VER	0.25	.969

Note: TAS = Tellegen Absorption Scale, DES = Dissociative Experiences Scale, ICMI = Inventory of Childhood Memories and Imaginings, VVIQ = Vividness of Visual Imagery Scale, VVQ VIS = Verbalizer-Visualizer Questionnaire – visual cognitive style, VVQ VER = Verbalizer-Visualizer Questionnaire – verbal cognitive style

**Hypothesis 4.** To analyze Hypothesis 4, moderated mediation was analyzed, with self-esteem moderating the impact of the experimental manipulation (vs. control) that should increase experienced distrust; the latter was a mediator of the impact of the MD on the imagination inflation effect. Model 7 from the templates available in the PROCESS 4.1 [133] software for SPSS [132] was used. Self-esteem was found not to moderate the effect of MD induction and MD experienced (i.e., the difference between MD post- and pretest), B = 0.05, *SE* = .37, *t* = 0.14, *p* = .892. The stronger MD experienced was not associated with a greater susceptibility to imagination inflation effect, B = 0.002, *SE* = .01, **t* *= −0.44, *p* = .661. The overall moderated mediation model was not supported, the index of moderated mediation < 0.01 (95% CI [−0.007, 0.003]. As zero is within the confidence intervals, the moderating effect of self-esteem on MD induction on the indirect effect via MD experienced is not confirmed. This means that Hypothesis 4 is not supported.

## Discussion

The objective of this study was to examine whether MD is related to the imagination inflation effect among people who are aware of the discrepancies between their own memories and what they have imagined. Our aim was also to establish whether the influence of MD on imagination inflation is moderated by traits termed disengagement from reality and to test whether MD mediates the relationship between self-esteem and imagination inflation.

When discussing the results, we would like to start by stating that we confirmed the negative effect of imagery on memory. That is, with regard to imagined events, we observed a significant increase in the confidence that an event occurred in the participant’s life compared to non-imagined, control events. This result confirms the findings of previous studies and meta-analyzes [6,62,134]. In the context of the cases presented in the Introduction, it supports the notion that it is possible to produce mental images that are similar on several levels, such as sensory-perceptual detail to real events, thus making the imagined event more familiar, accessible and believable, which risks accepting of the imagined event as real [135].

The hypothesis of a correlative relationship between the two aspects of MD and susceptibility to the imagination inflation effect was partially confirmed. This hypothesis had two parts, regarding omission and commission errors. As we expected, there was no relationship between subjectively experienced susceptibility to make memory omission errors and susceptibility to the imagination inflation effect. This confirms that errors of omission are not directly related to the imagination inflation effect. In the procedure used in the study, imagining events may cause confusion between real and imagined events (e.g., filling memory gaps with imagined details) or misattribution of the source, but not “erasure” of real events from memory (however, suggesting that an event was not experienced can lead to omission errors (see for review [17])). Thus, the subjective belief in one’s own tendency to forget does not interfere with the acquisition of new “memories” as a result of imagining certain events.

Contrary to our prediction, the correlation between subjectively perceived susceptibility to make commission errors and susceptibility to the imagination inflation effect was also non-significant. Researchers have more recently distinguished between the aspect of committing errors in the MD trait, which was previously treated uniformly [17], and as a result, there is relatively few research on its role in the development of memory distortions. In one of them, regarding non-believed memories, it has been shown that people perceiving themselves as being more likely to have commission errors’, compared to those with a low tendency, had a greater tendency to form spontaneous non-believed memories [85]. However, there is an important difference between non-believed memories and imagination inflation effect evoked in our study: the first one involves a process of critical re-evaluation of a once-believed memory, which may lead to reduced belief in occurrence for the event [136], while imagination inflation involves the acceptance of an imagined event as real without such critical analysis. With non-believed memories, the person realizes that the memory may be wrong. In our study, a person has no initial distrust because they do not realize that the memory comes from the imagination.

Another explanation of the nonsignificant correlation between MD measured by MDS and imagination effects may be based on the distinction between “belief-in-occurrence” and “memory” [137]. Imagination inflation refers to “the phenomenon that imagining a low probability childhood event promotes subjective confidence that the event actually happened” [9]. So, this phenomenon is about increasing one’s belief that an imaginary event took place, which does not always translate into completely false memories. The MD measures the one’s tendency to doubt “that their successful recollections of past events are reliable or accurate” [17]. Most of the MDS items pertains to doubting memory accuracy (e.g., seeking external confirmation of a memory). And susceptibility to imagination inflation effect is not typically on doubting memory, but rather about the erroneous belief about (false) memories.

The hypothesis regarding the effect of MD on susceptibility to the imagination inflation effect, which should be revealed in groups sensitized to discrepancies, was also not confirmed. Although MD manipulation was effective (i.e., confidence in the experimental group decreased after it was applied), participants in the experimental group who received the cue(s) were no more susceptible to the imagination inflation effect than participants in the control group who received the cue(s). However, the manipulation of the memory distrust that we used in this study was not focused on questioning the degree of belief in the occurrence of events in a person’s life. Rather, it was a general manipulation that undermined the perceived ability to recall past events and to recall learned material. Therefore, it is possible that participants’ confidence only in this type of memory was undermined, which did not translate into performance in the imagination inflation task.

Additional analysis of the effect of cues on the effect imagination inflation depending on whether MD was induced provided valuable information. The first one relates to the reduction in susceptibility to the imagination inflation effect following the administration of two (but not one) cues in the group without MD induction. This effect replicates an earlier study by Sherman et al. [35] reporting that two cues can reduce susceptibility to the imagination inflation effect. The second relates to the MD induction group, in which no beneficial effect of cues on the imagination inflation effect was reported. Perhaps when participants were induced into a state of MD, the effect of the cues was “blocked”. This is an effect worth further exploration.

None of the hypotheses regarding the moderating role of traits relating to disengagement from reality on the influence of MD on imagination inflation, as well as the hypothesis stating that MD mediates the relationship between self-esteem and imagination inflation were confirmed. The lack of an expected effect may be attributed to the nature of the experimental manipulation. The decrease in trust in one’s own memory was significant, allowing us to consider the manipulation effective . However, participants still maintained trust in their memory at almost 53% after manipulation. Therefore, it is difficult to conclude that we induced a state that Gudjonsson refers to as ‘profound distrust’ toward memory. It may be that the effects start to emerge at much higher levels of MD than those induced in our study (and for ethical reasons in studies of this type, in general). Likewise, the MD trait may be highly exacerbated and related to the development of memory distortions only in a very small group of people (e.g., those suffering from obsessive-compulsive disorder), which we were unable to reach when studying the general population.

### Limitations of the study and future directions

We consider the choice of experimental paradigm and the way MD was induced to be the major limitation of the study conducted. We decided to use the Garry et al. paradigm [6] because we wanted to use the same methodology that was used in the first study investigating the relationship between MD and the imagination inflation effect [138]. As mentioned in the Introduction, in this paradigm, it is not possible to control awareness of discrepancies between what is true and what is suggested to participants, which is crucial to our assumptions about how MD works. Future research could use the paradigm created by Goff and Roediger [8], in which participants are presented with simple action statements (e.g., “fold the piece of paper”) and in some conditions, they perform the actions, while in others, they only imagine performing them. After a delay, participants imagine performing certain actions multiple times (e.g., once, three times, or five times). Some of these actions are actions from the first session that participants may have either performed, imagined, or heard, and some actions are completely new. During the last phase, participants complete a recognition test in which they should recognize action statements only if they encountered them in the first session and then indicate whether those action statement was carried out, imagined, or only heard. It should be noted, however, that this paradigm is strictly concerned with the formation of false memories, whereas the paradigm of Garry et al., [6] is concerned with increasing the confidence that an event occurred in a person’s life (it may or may not be a false memory) [8]. Moreover, it does not concern autobiographical childhood events, but rather actions.

As far as experimental manipulation is concerned, we used a manipulation already used in previous studies [18]. Here, manipulation also lowered trust in memory, which was its purpose. However, the manipulation was not related to the task content, so it may not have been effective in relation to the memory of these specific events. How important it is to induce distrust for a specific memory is shown by research on the powerful effect of interrogative suggestibility, the whole procedure of which involves challenging the participant’s response to the presented experimental material [104]. Similarly, Zhang et al. [139] successfully manipulate feedback on performance in the memory test in non-believed memories research by relating memory questioning to specific action/person from the experimental task. In future studies, therefore, the manipulation could be modified to be more ecological, i.e., related to the imagined events.

In conclusion, future research further exploring the relationship between MD and the imagination inflation effect could use the paradigm of eliciting false memories over which the researcher has control (such as Goff and Roediger [8]) and elicit MD, e.g., by showing “proof” that the memory is false (e.g., [140]). Furthermore, using behavioral measures in such a study, e.g., reaction time, it could be possible to try to elucidate the mechanisms of such false memories (memory erasure vs*.* non-memory mechanisms, e.g., conformity) [141,142].

It may be interesting to consider and research how the results of the present research relate to phenomena similar to imagination inflation, like observation inflation [143]. In this case, merely observing the actions of others may lead to false memories of having performed those actions yourself. It may be interesting to analyze whether memory distrust is linked to susceptibility to the observation inflation effect. This is possible because severe memory distrust can cause uncertainty as to whether one actually performed the action or merely observed it. Temporal distance may be a moderator of this relationship. If the time interval between observing an action and reporting whether one performed it is large, memory distrust may be more important than when the interval is short.

Similarly, memory distrust may be related to episodic counterfactual thinking, that is, imagining alternative ways in which past personal events, which truly happened could have occurred (review: [144]). This phenomenon may be related to obsessive compulsive disorder which in turn is connected to memory distrust [24]. Studying relationships between memory distrust and the tendency to counterfactual thinking may be an interesting future direction.

## Supporting information

S1 File Details of the statistical models. (PDF)

S2 File Scatterplots of the relation between memory distrust and the index of susceptibility to the imagination inflation effect. (PDF)

S3 File Power analysis. (PDF)
