# Supplementary material for: Memory distrust and imagination inflation: A registered report
Source: PLoS One. 2025 Aug 1;20(8):e0327638. doi: 10.1371/journal.pone.0327638 (PMC12316254; doi:10.1371/journal.pone.0327638)
Supplement: S1 File — (PDF) [file pone.0327638.s001.pdf]

Details of the Statistical Models

S1 Table 1. Generalized Estimating Equations: dependent variable: LEI difference, predictors: Induction of memory distrust, Sensitization, Item imagining

	Wald $chi^2(1)$	*p*
(Constant)	52.71	<.001
Induction of memory distrust	0.65	.422
Sensitization present or absent	0.01	.926
Item imagined or not	23.43	<.001
Induction of memory distrust × Sensitization present or absent	6.68	.010
Induction of memory distrust × Item imagined or not	0.73	.393
Sensitization present or absent × Item imagined or not	1.76	.185
Induction of memory distrust × Sensitization present or absent × Item imagined or not	0.59	.442

S1 Table 2. Generalized Estimating Equations: dependent variable: LEI difference, predictors: Induction of memory distrust, Number of sensitization cues, Item imagining

	Wald $chi^2(1)$	*p*
(Constant)	60.73	<.001
Induction of memory distrust	0.16	.690
Item imagined or not	26.70	<.001
Number of sensitization cues	0.08	.959
Induction of memory distrust × Item imagined or not	0.38	.540
Induction of memory distrust × Number of sensitization cues	8.77	.012
Item imagined or not × Number of sensitization cues	2.53	.283
Induction of memory distrust × Item imagined or not × Number of sensitization cues	0.97	.615
