# Supplementary material for: Memory distrust and imagination inflation: A registered report
Source: PLoS One. 2025 Aug 1;20(8):e0327638. doi: 10.1371/journal.pone.0327638 (PMC12316254; doi:10.1371/journal.pone.0327638)
Supplement: S2 File — (PDF) [file pone.0327638.s002.pdf]

Scatterplots of the Relation Between Memory Distrust and the Index of Susceptibility to the Imagination Inflation Effect

S2 Fig 1. Scatterplots of the Relation Between SSMQ and the Index of Susceptibility to the Imagination Inflation Effect for Subgroups

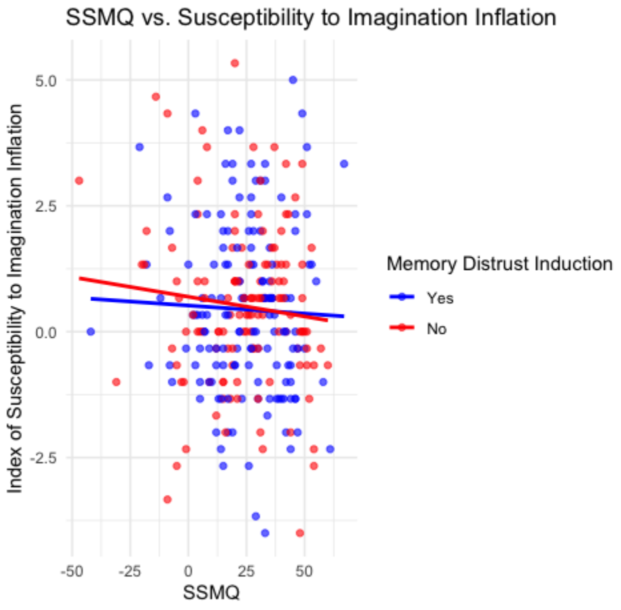

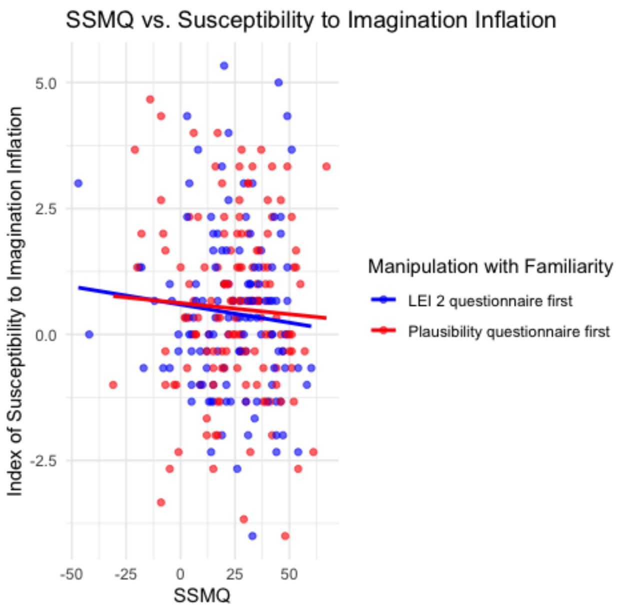

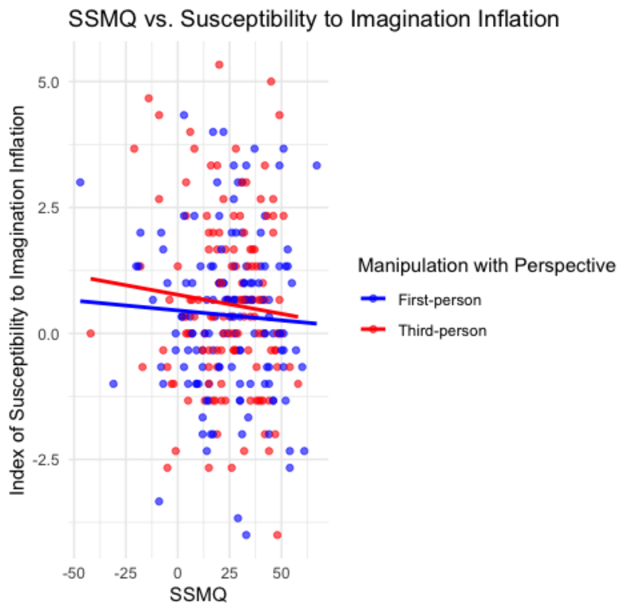

S2 Fig 2. Scatterplots of the Relation Between MDS and the Index of Susceptibility to the Imagination Inflation Effect for Subgroups

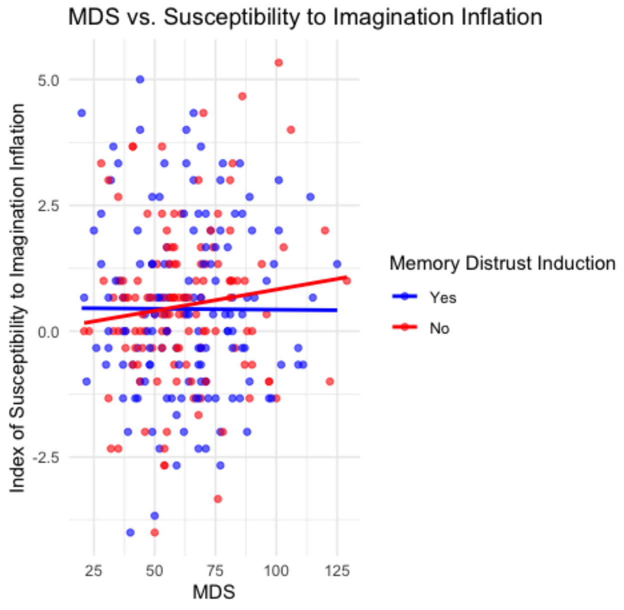

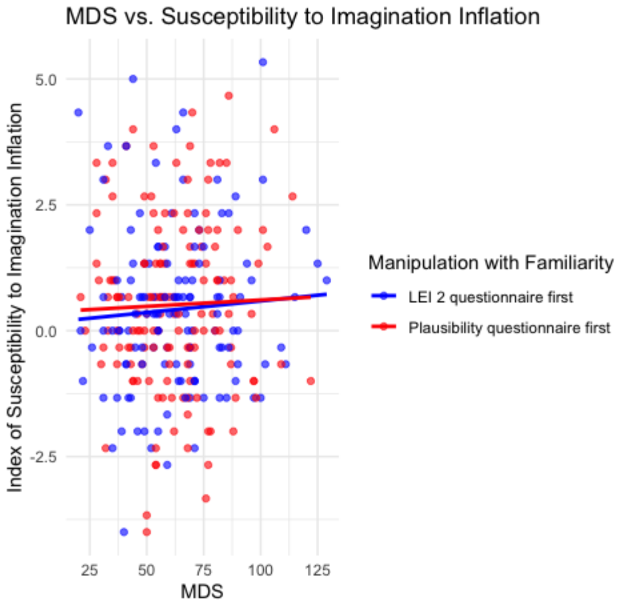
MDS vs. Susceptibility to Imagination Inflation
Index of Susceptibility to Imagination Inflation
5.0
2.5
0.0
-2.5
25
50
75
100
125
MDS
Manipulation with Familiarity
LEI 2 questionnaire first
Plausibility questionnaire first

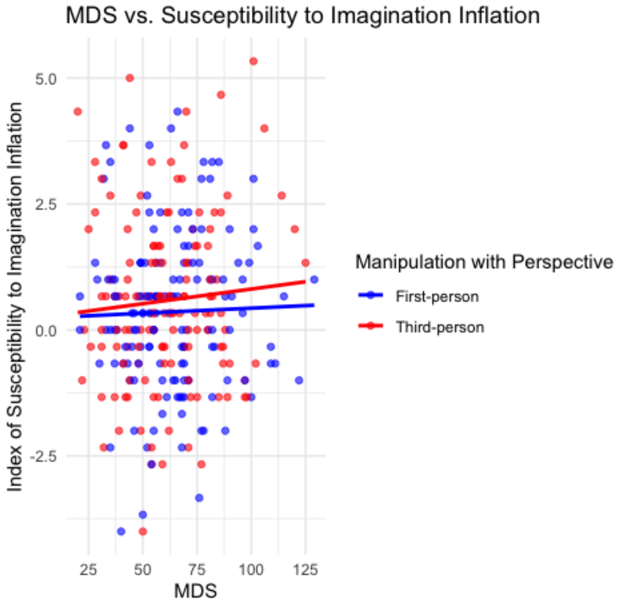
MDS vs. Susceptibility to Imagination Inflation
Index of Susceptibility to Imagination Inflation
5.0
2.5
0.0
-2.5
25
50
75
100
125
MDS
Manipulation with Perspective
First-person
Third-person
