# Supplementary material for: Memory distrust and imagination inflation: A registered report
Source: PLoS One. 2025 Aug 1;20(8):e0327638. doi: 10.1371/journal.pone.0327638 (PMC12316254; doi:10.1371/journal.pone.0327638)

Power Analysis

ANOVA Interaction large effect

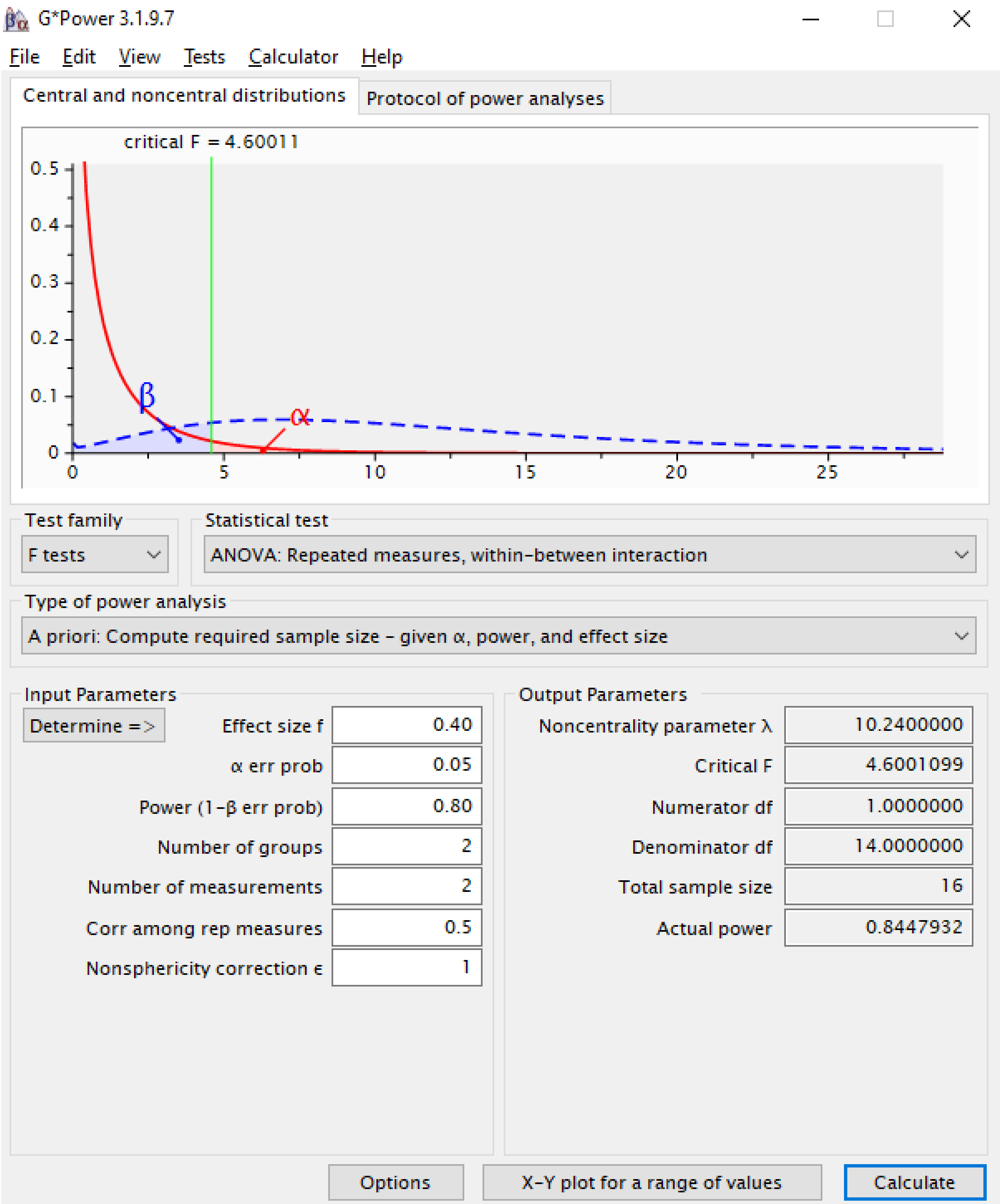

ANOVA Interaction medium effect

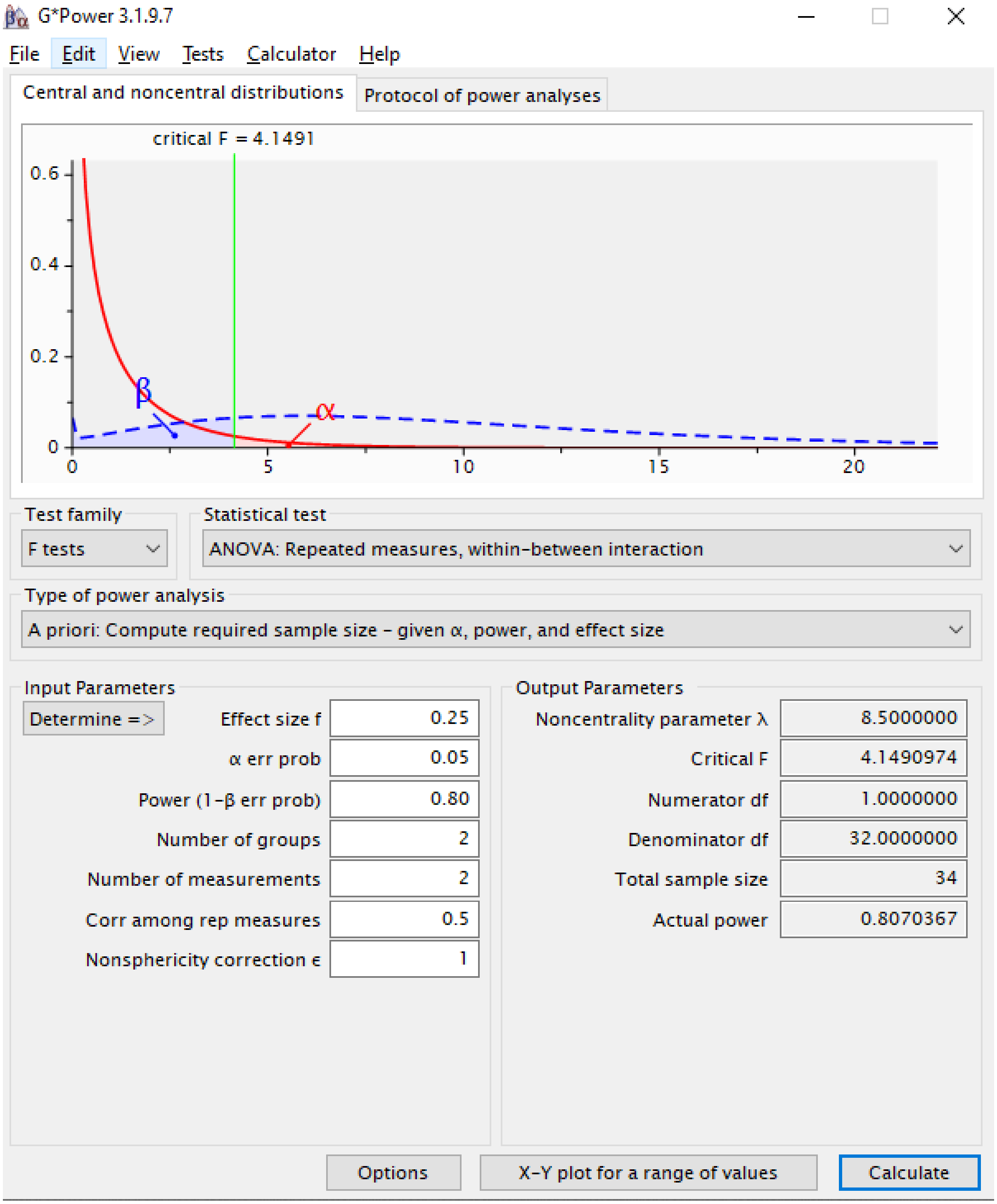

ANOVA Interaction small effect

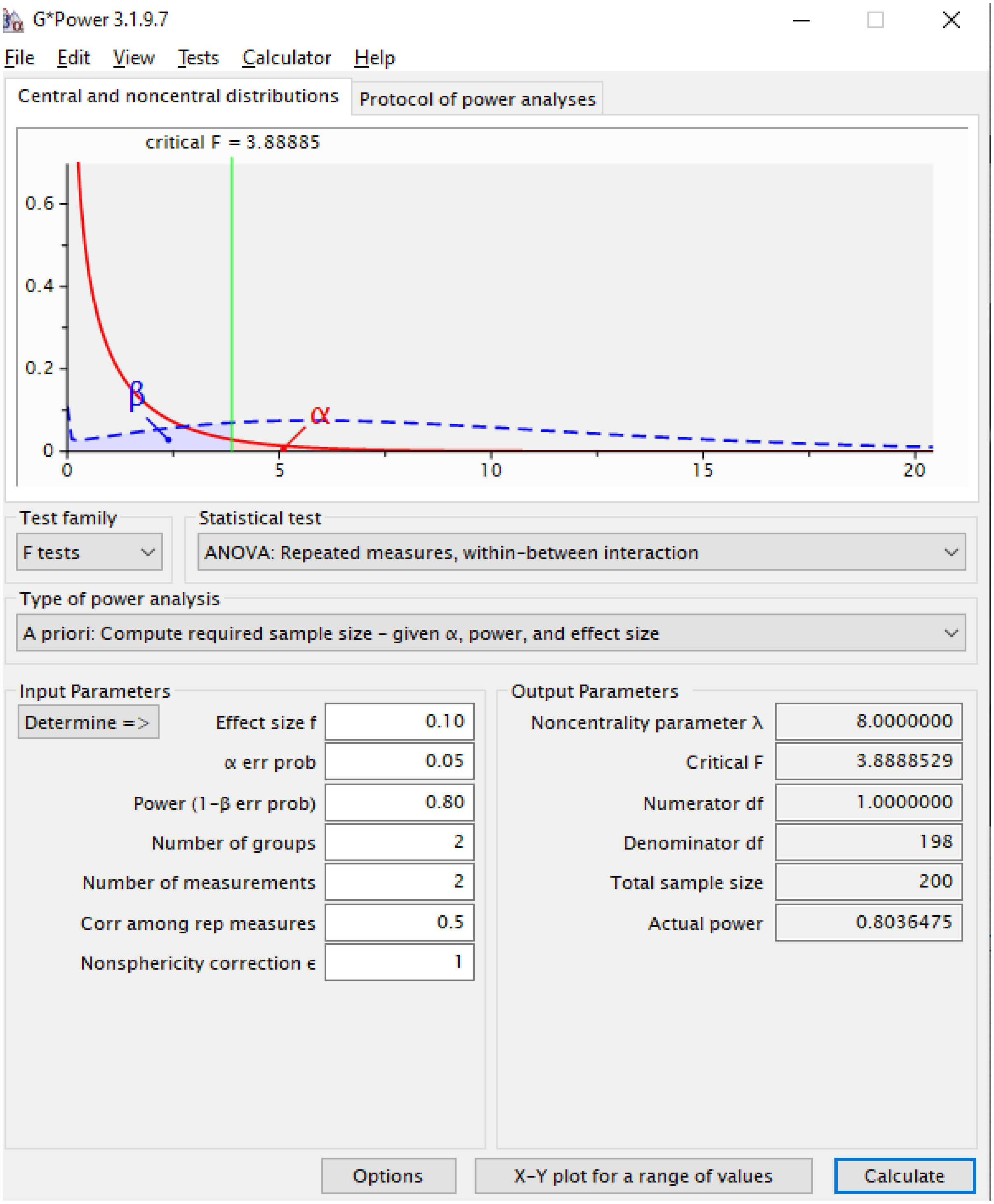

Moderation large effect

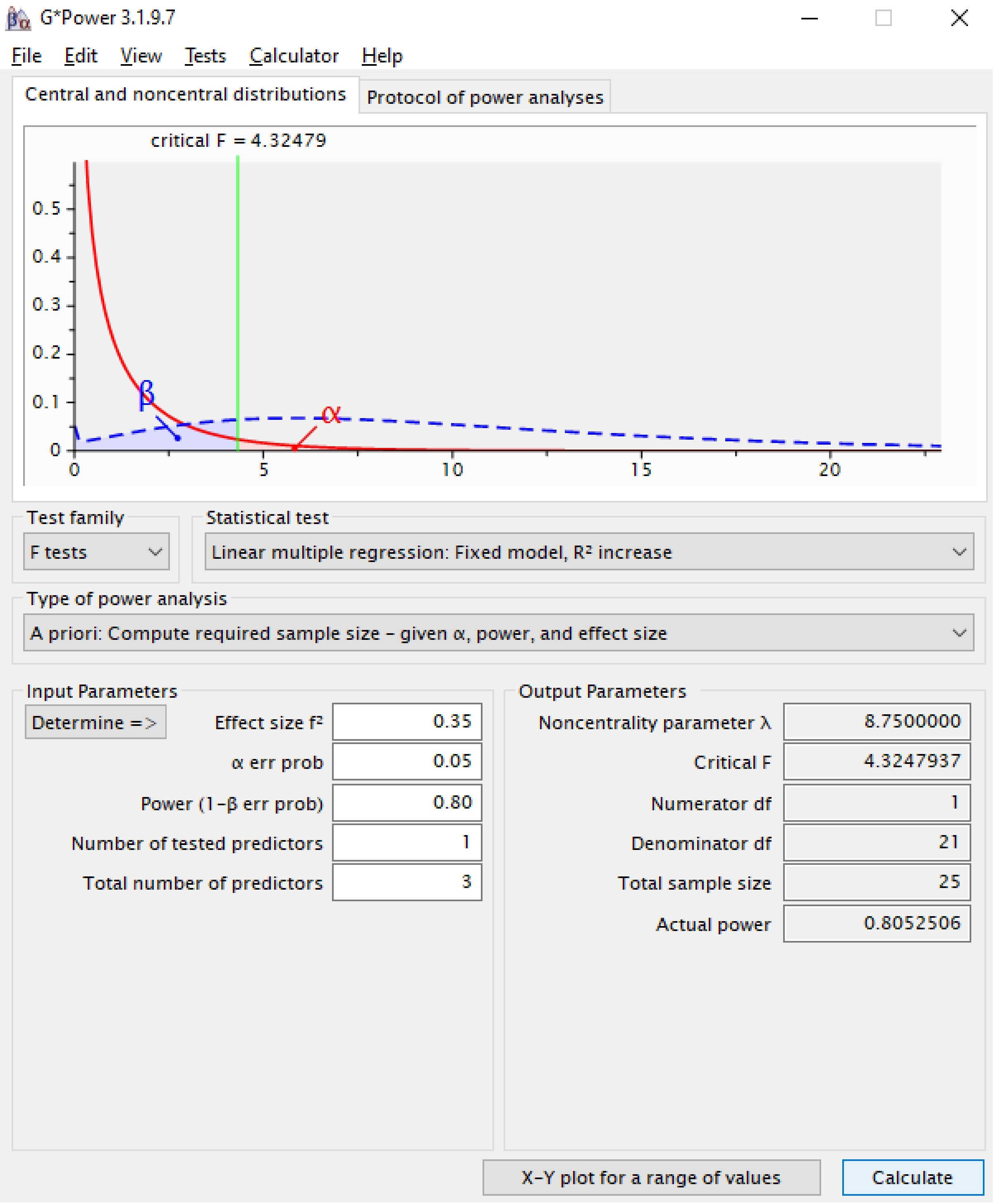

Moderation medium effect

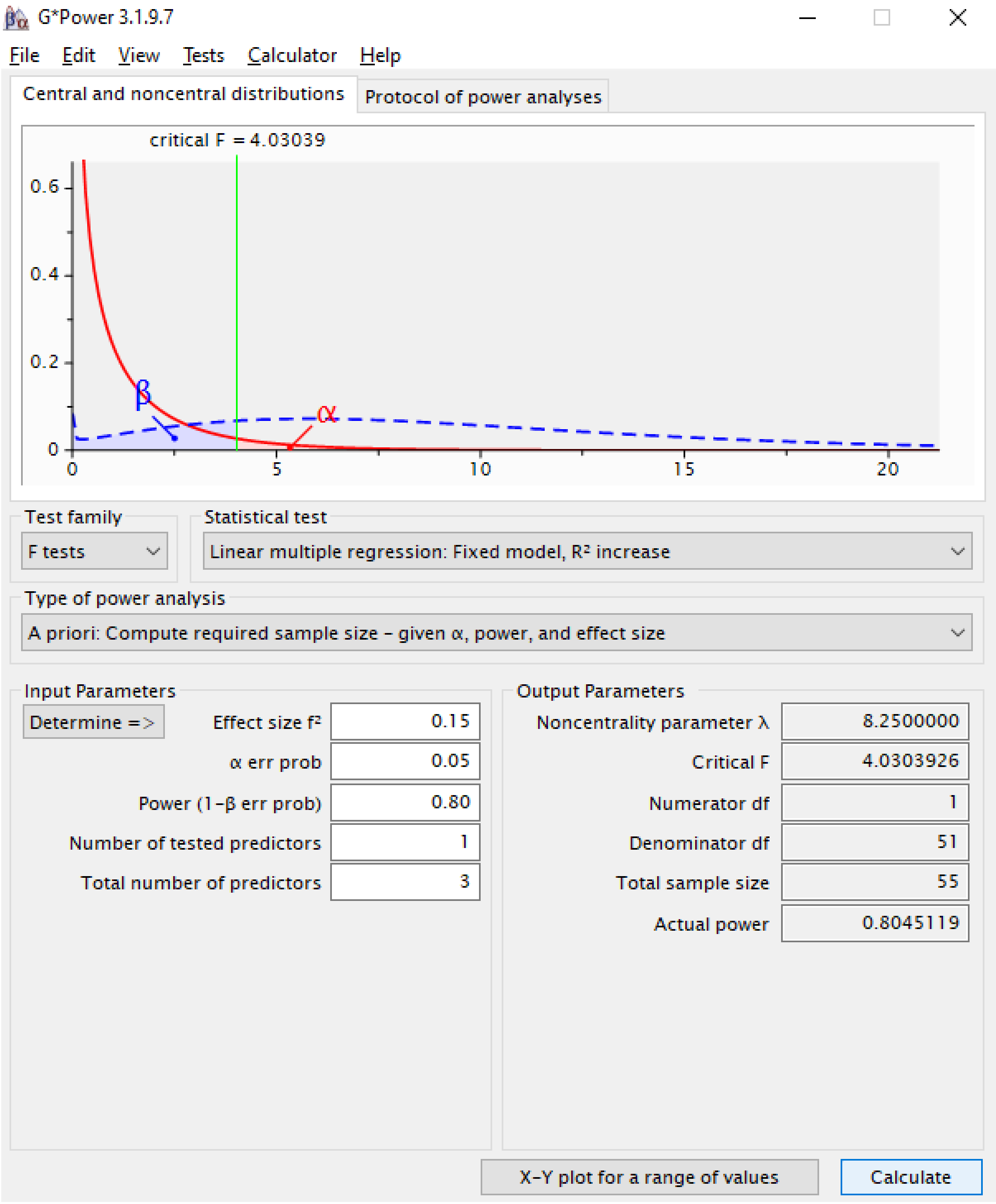

Moderation small effect

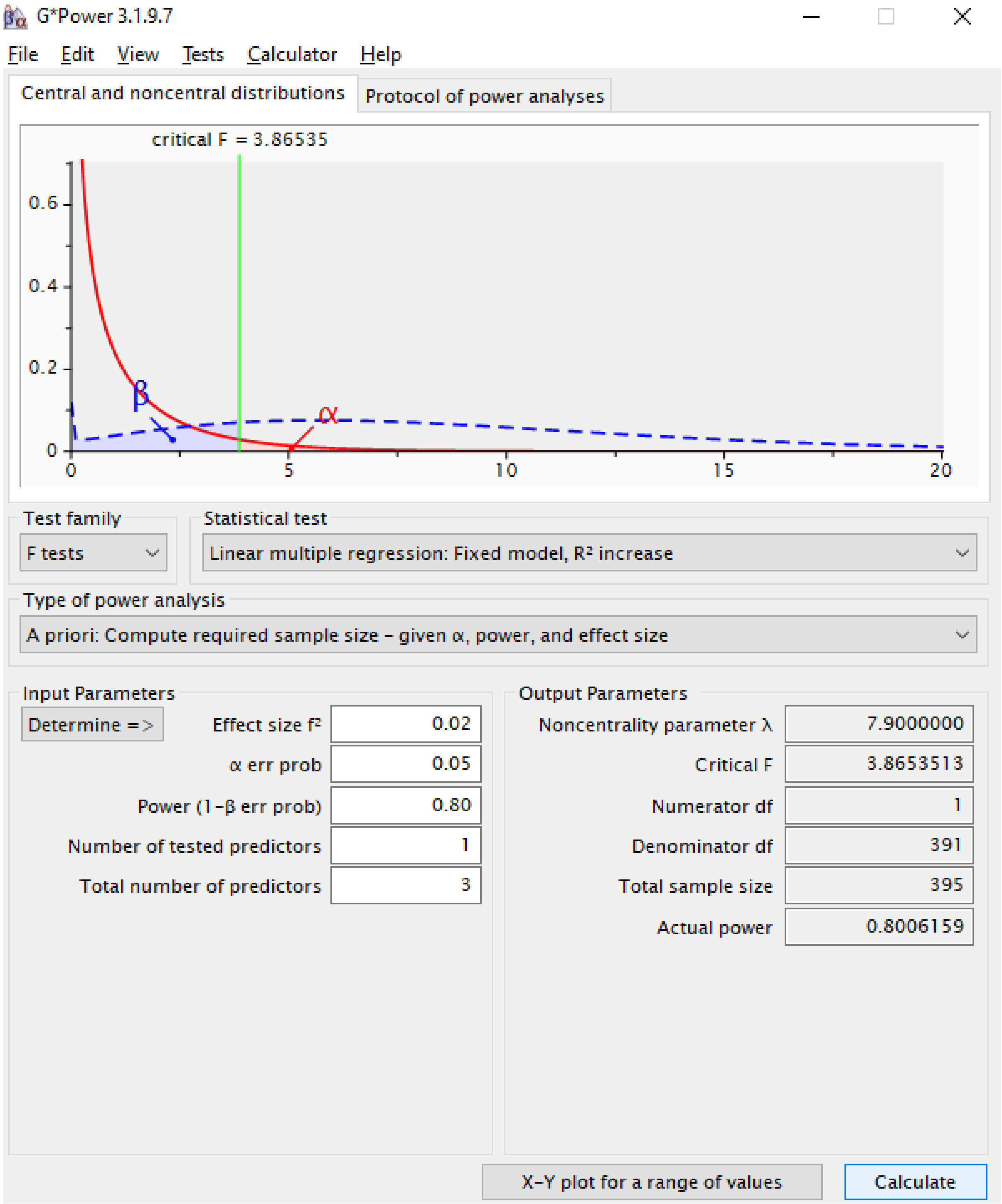

Repeated ANOVA large effect

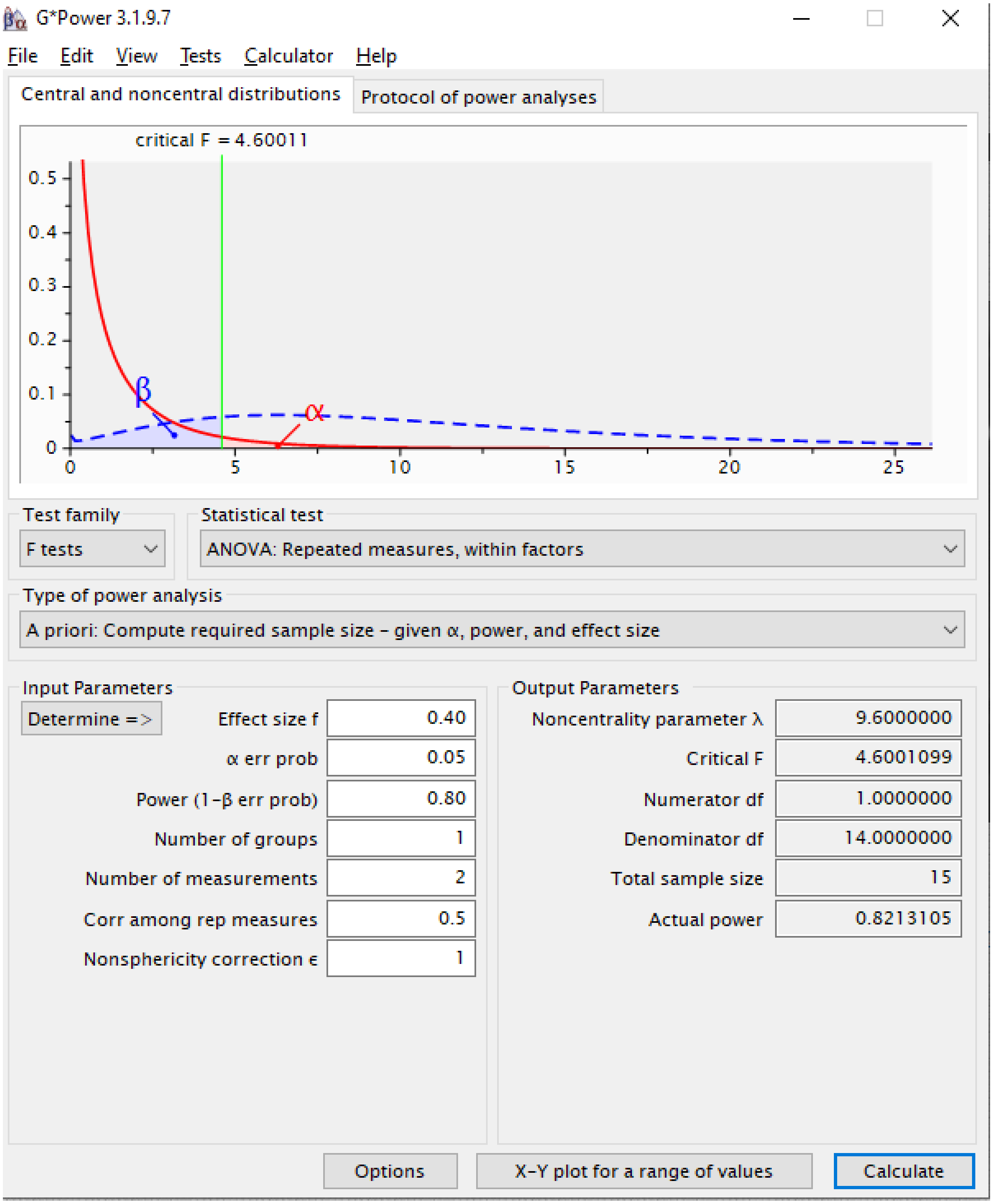

Repeated ANOVA medium effect

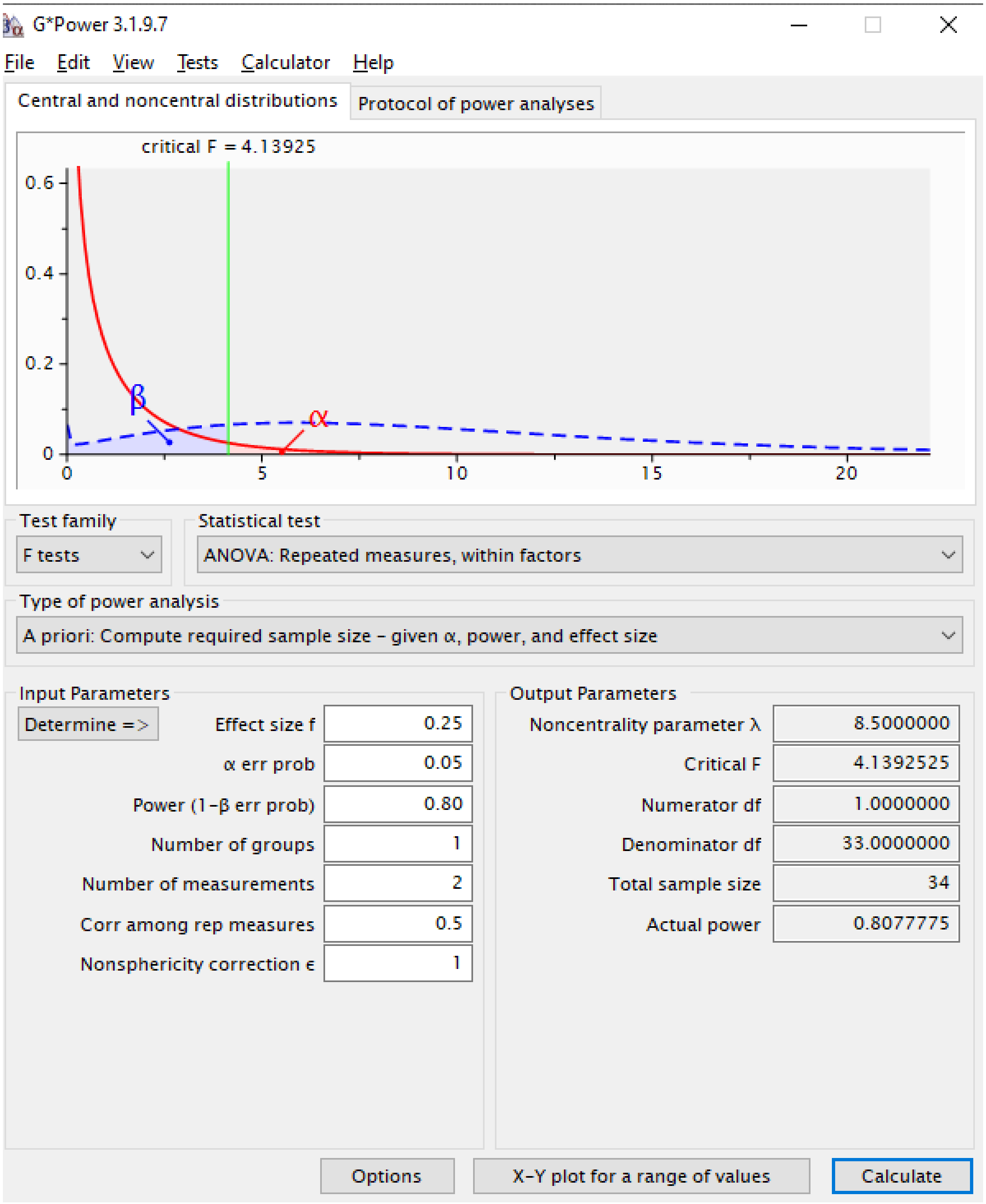

Repeated ANOVA small effect

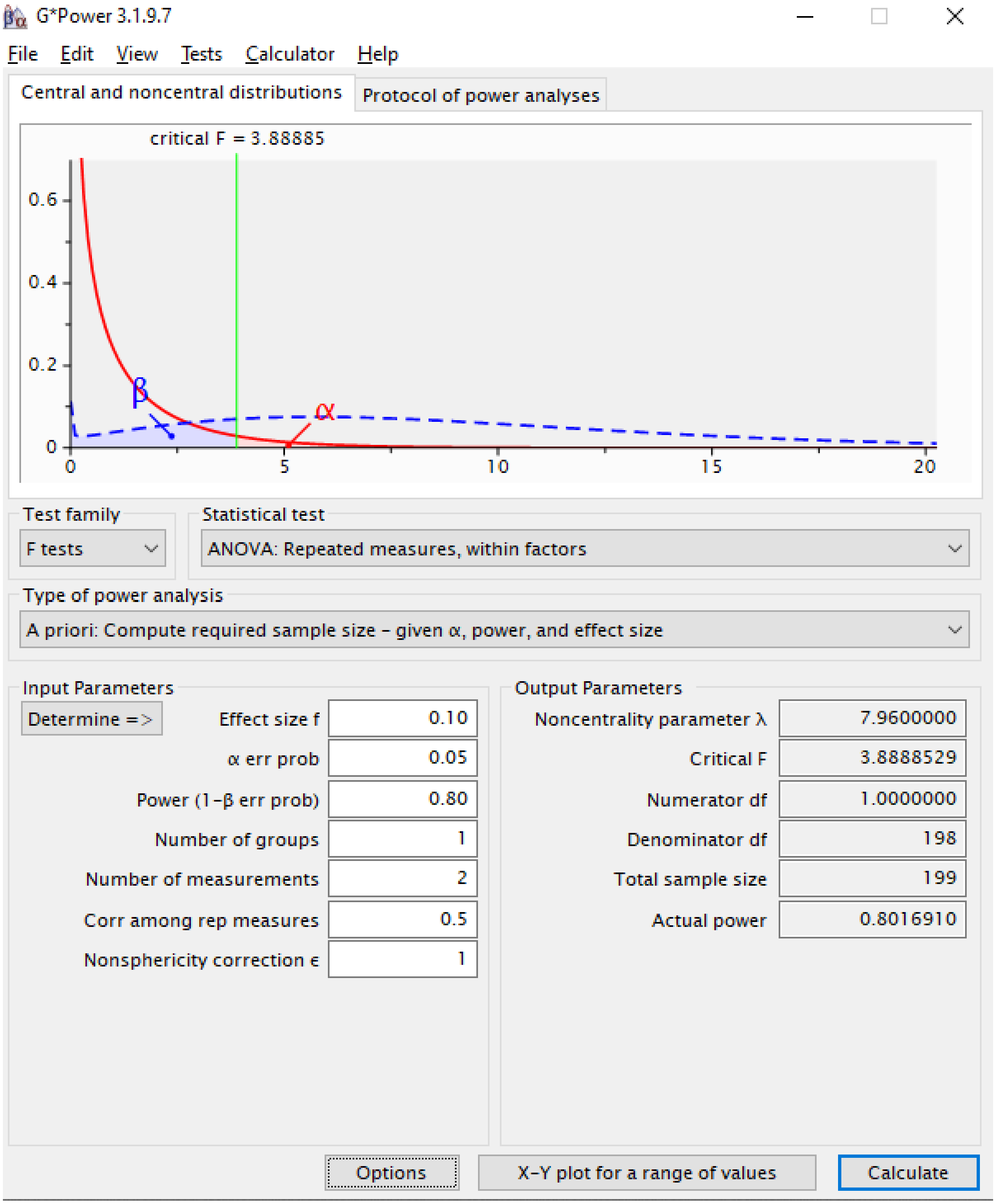

Supplement: S3 File — (PDF) [file pone.0327638.s003.pdf]
